# Liver Tumor Segmentation with Deep Learning: A Comparative Analysis of CNN-, Transformer-, and YOLO-Based Models on the ATLAS MRI

**DOI:** 10.3390/diagnostics16050649

**Published:** 2026-02-24

**Authors:** Büşra Karabağ, Kubilay Ayturan, Fırat Hardalaç

**Affiliations:** Department of Electrical and Electronics Engineering, Gazi University, 06570 Ankara, Turkey; busra.karabag1@gazi.edu.tr (B.K.); firat@gazi.edu.tr (F.H.)

**Keywords:** hepatocellular carcinoma, liver tumor segmentation, deep learning, MRI, transformer-based segmentation, volumetric segmentation

## Abstract

**Background/Objectives:** Hepatocellular carcinoma (HCC) remains a leading cause of cancer-related mortality worldwide, where accurate liver and tumor segmentation from magnetic resonance imaging (MRI) is essential for diagnosis, treatment planning, and disease monitoring. Despite recent advances, MRI-based segmentation remains challenging due to data heterogeneity and limited annotated datasets. This study aims to systematically compare convolutional, transformer-based, and detection-based deep learning approaches for liver and HCC segmentation using contrast-enhanced MRI. **Methods:** A comprehensive evaluation was conducted on the ATLAS MRI dataset, including 2D- and 3D-CNN, transformer-based architectures, and single-stage YOLO-based segmentation frameworks. All models were trained using consistent preprocessing, patient-level data splits, and standardized evaluation metrics, including Dice coefficient, Intersection over Union (IoU), precision, recall, and F1-score. **Results:** Volumetric convolutional models achieved the highest segmentation accuracy, with the 3D nnU-Net yielding superior performance for both liver (Dice: 0.946) and tumor (Dice: 0.892) segmentation. Transformer-based models demonstrated competitive results, particularly in capturing global contextual information and improving boundary delineation, while YOLO-based approaches provided balanced accuracy with substantially reduced computational cost. **Conclusions:** The findings confirm that volumetric CNNs remain the most accurate solution for MRI-based liver and HCC segmentation, whereas transformer- and YOLO-based frameworks offer complementary advantages for specific clinical and resource-constrained scenarios.

## 1. Introduction

HCC is the most prevalent form of primary liver cancer and remains one of the leading causes of cancer-related mortality worldwide [1]. In recent years, advances in deep learning-based image analysis have significantly improved the automation and precision of liver tumor segmentation, particularly in computed tomography (CT) imaging [2,3,4].

Although CT imaging has traditionally dominated liver tumor segmentation research because of its widespread clinical adoption, the generalizability of these models to MRI remains limited due to modality-specific contrast behavior and noise characteristics [4]. Numerous studies have demonstrated the effectiveness of U-Net and its variants, including ResUNet and Mask R-CNN-based frameworks, in CT imaging [5,6,7,8,9,10,11,12]. While these approaches achieve high accuracy in CT scans, their performance does not directly translate to magnetic resonance imaging (MRI), where imaging characteristics, contrast dynamics, and noise distributions differ substantially.

MRI offers superior soft-tissue contrast and multiphase imaging capabilities, making it particularly valuable for early tumor detection and precise boundary delineation. However, MRI-based liver tumor segmentation has received comparatively less attention due to the limited availability of annotated datasets and the increased heterogeneity of MRI acquisitions [13,14,15]. The recently introduced ATLAS dataset [16] addresses this limitation by providing expert-annotated, contrast-enhanced MRI scans specifically designed for HCC treatment planning. Prior studies on ATLAS have shown that volumetric CNNs and hybrid CNN–Transformer architectures can achieve promising results, especially for small and low-contrast tumors [17].

Conventional CNNs effectively capture local features but are limited in modeling long-range dependencies; transformer-based models address this via self-attention and global context modeling [18,19,20]. Models such as DynTransNet, Swin-Transformer, and TransUNet have demonstrated improved boundary awareness and robustness on heterogeneous MRI data, making them strong candidates for liver tumor segmentation on ATLAS [17,18,19,20].

In parallel, single-stage detection-based frameworks such as YOLO have emerged as efficient alternatives for real-time segmentation tasks. Although originally designed for object detection, recent YOLO variants offering favorable trade-offs between accuracy and computational efficiency [21]. However, direct and systematic comparisons between CNN-based, transformer-based, and YOLO-based approaches on the same MRI dataset remain limited.

Therefore, this study addresses the following gaps in the literature:

- The lack of comprehensive comparisons of CNN-, transformer-, and YOLO-based segmentation paradigms on contrast-enhanced MRI;
- The limited evaluation of transformer-based architectures on the ATLAS dataset under standardized protocols;
- The absence of analysis balancing segmentation accuracy and computational efficiency for potential clinical deployment.

Beyond establishing a performance benchmark, the study employs an explicit deployment-oriented evaluation framework, a significant development in the field. The following section provides a concise summary of the novel contributions of the present work.

- Deployment-Oriented Selection Guide: A clinical dichotomy for AI model selection in MRI analysis is hereby defined, with YOLO-based frameworks being positioned as optimal tools for rapid triage and screening (>85 FPS) in resource-constrained settings. Volumetric 3D nnU-Net is established as the gold standard for high-precision preoperative planning, where computational cost is secondary to boundary accuracy.
- Volumetric Necessity in MRI: By systematically analyzing failure cases, we demonstrate that 2D architectures (U-Net, ResUNet) fundamentally fail to detect small HCC lesions due to the lack of inter-slice spatial continuity. We provide analytic evidence that volumetric context is not optional but mandatory for reliable small-tumor detection in MRI, distinguishing it from CT-based tasks where 2D models often suffice.
- Unified Evaluation Protocol Across Paradigms: This study represents one of the significant contributions to the literature by directly comparing single-stage detection models (YOLOv8/v11) against volumetric segmentation networks on the same voxel grid basis. This methodological integration facilitates the direct measurement of the accuracy-efficiency balance adapted to ATLAS MR data.

To this end, we present a systematic and unified evaluation of 2D and 3D CNNs, transformer-based models, and YOLO-based segmentation frameworks on the ATLAS MRI dataset, using consistent preprocessing, patient-level data splits, and standardized performance metrics. These analyses aim to clarify the complementary strengths and limitations of deep learning paradigms for MRI-based segmentation. Beyond establishing a performance benchmark, this study explicitly addresses the trade-off between inference speed and volumetric precision in MRI analysis. Unlike prior works that isolate these architectures, we position YOLO-based models (real-time efficiency) against volumetric nnU-Net frameworks (surgical precision) to define a clinical selection guide. This analysis provides evidence-based recommendations on which architecture suits specific clinical scenarios—ranging from rapid triage and screening in resource-constrained settings to high-precision preoperative planning.

## 2. Materials and Methods

### 2.1. Dataset Description

The ATLAS dataset (France) [16,22] was used for liver and tumor segmentation from contrast-enhanced magnetic resonance imaging (CE-MRI). The ATLAS dataset comprises contrast-enhanced MRI volumes from 60 patients diagnosed with HCC.

The dataset includes images acquired at different post-contrast phases, reflecting real-world clinical heterogeneity. Specifically, 33 patients were scanned in the arterial phase, 10 in the venous phase, eight in the late phase, seven with unknown contrast phase, and two without contrast enhancement. This phase diversity enables evaluation of model robustness under varying imaging characteristics and contrast dynamics.

All data were partitioned at the patient level using a 70/15/15 split for training, validation, and testing, respectively. Strict patient-level separation was explicitly enforced to prevent data leakage; this ensures that no slices from a patient in the training set ever appear in the validation or test sets. Consequently, the test set functions as a completely independent, held-out external cohort. Stratified sampling based on tumor volume was applied to ensure balanced representation across subsets. This ensured that small (<500 mm^3^), medium, and large tumors were equally represented across all subsets to prevent size-related bias.

To provide a detailed quantitative assessment of the tumor burden distribution, volumetric statistics (mean, median, and range) for the training, validation, and test sets are summarized in Table 1. As shown, the tumor volumes exhibit a wide range, spanning from small early-stage lesions to large advanced carcinomas. The consistent distribution of mean and median values across splits confirms that the stratified sampling strategy successfully prevented size-related bias between the subsets.

In parallel with volume-based stratification, the data partitioning strategy also aimed to preserve the distribution of contrast phases. Given the predominance of the arterial phase (55%) in the dataset, the training, validation, and test splits were curated to maintain a proportional representation of arterial, venous, and late-phase acquisitions. This approach ensures that the models are exposed to the distinct contrast dynamics of each phase during training while being evaluated on a representative mix of scanning protocols. The distribution of contrast phases according to data sections is shown in detail in Table 2.

Clear and transparent reporting of dataset partitioning is critical in medical image segmentation, as improper splits can lead to biased performance estimates and limited generalizability. Prior studies have emphasized that reproducibility and fairness of evaluation metrics such as Dice, IoU, precision, and recall strongly depend on how data subsets are defined [23,24,25]. In particular, Isensee et al. demonstrated that careful patient-level splitting and cross-validation are essential for stabilizing performance across heterogeneous medical imaging datasets [23].

To ensure unbiased evaluation, the independent test set was excluded from all training and cross-validation procedures and accessed only once for final performance reporting.

**Figure 1:**
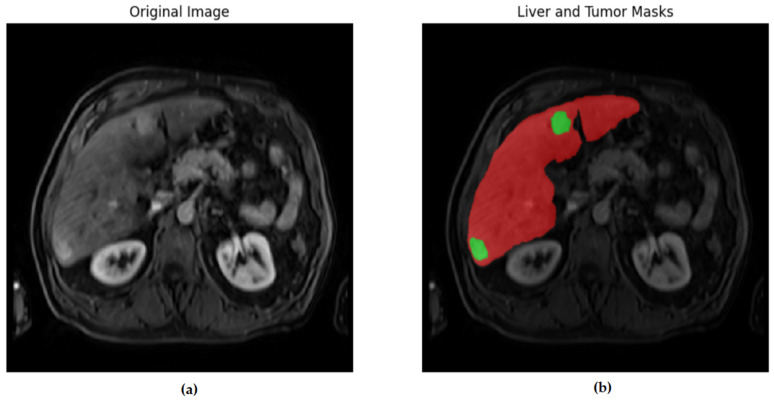
(**a**) Unlabeled liver image; (**b**) labeled liver image.

**Figure 2:**
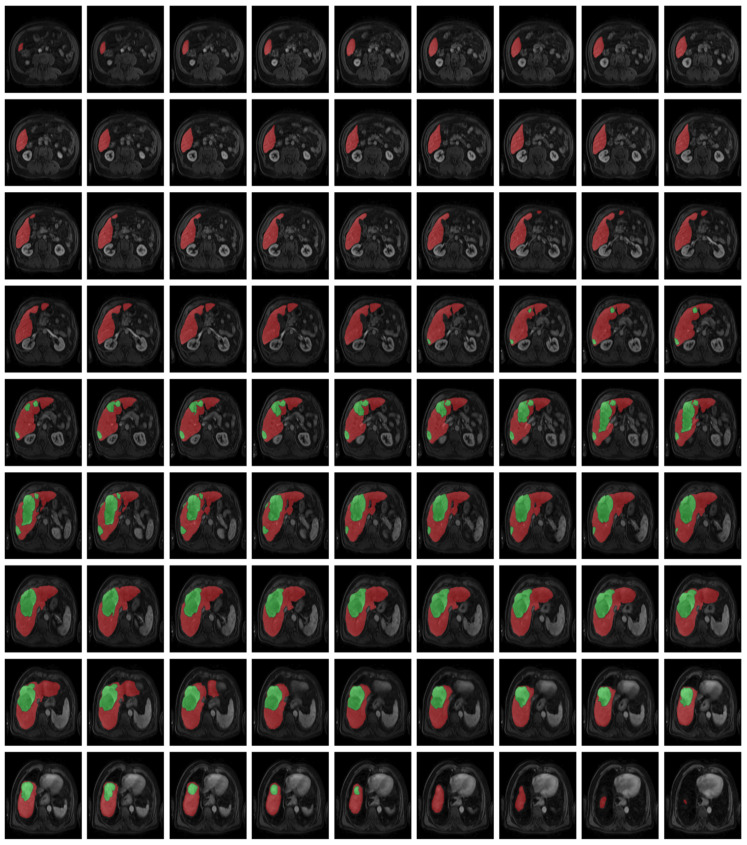
All liver sections from one patient.

**Figure 3:**
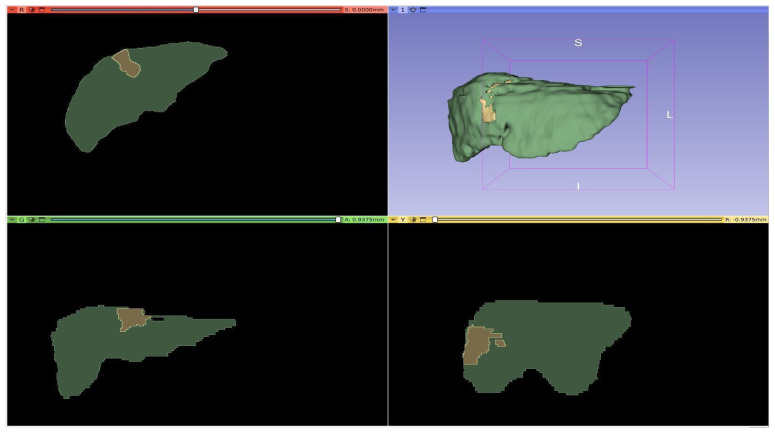
A cross-section of patient data in axial, sagittal, coronal planes and a 3D liver image in 3D Slicer.

**Figure 4:**
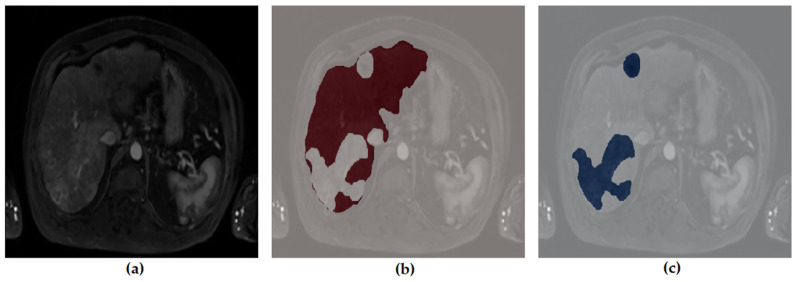
(**a**) Unlabeled image, (**b**) liver mask, and (**c**) tumor mask.

Figure 1, Figure 2, Figure 3 and Figure 4 illustrate representative MRI slices, volumetric liver anatomy, and the corresponding liver and tumor masks used as ground truth annotations.

The unlabeled image (Figure 1a) represents the raw contrast-enhanced MRI scan, while the labeled image (Figure 1b) includes the manual segmentation masks for liver and tumor regions. This figure highlights the role of expert-annotated ground truth in providing reliable training and validation data for deep learning-based segmentation models.

Figure 2 provides a patient-level visualization of axial liver slices, emphasizing the volumetric continuity leveraged by 3D segmentation models.

Figure 2 presents the complete set of liver slices from a single patient in the ATLAS dataset. Displaying all sections together allows for the visualization of liver morphology across the axial axis and demonstrates the dataset’s volumetric nature, which is essential for 3D segmentation models.

The images were analyzed in detail in axial, sagittal, and coronal planes and three dimensions (3D) using the 3D Slicer application.

Figure 3 depicts multi-plane visualizations (axial, sagittal, coronal) together with a 3D reconstruction of the liver obtained from the MRI data.

Figure 3 illustrates multi-plane visualization of a patient’s MRI data, including axial, sagittal, and coronal sections, as well as a reconstructed 3D liver model generated in 3D Slicer. This figure highlights the richness of volumetric imaging and the necessity of 3D approaches for accurate organ and tumor delineation.

In addition, the liver and tumor masks created during the pre-processing phase were visualized separately to verify that they accurately represent the anatomical structures. Figure 4 illustrates the preprocessing stage, showing the original MRI image alongside the generated liver and tumor masks.

Figure 4 demonstrates the pre-processing stage used to generate segmentation masks. The unlabeled input image (Figure 4a) is shown alongside its corresponding liver mask (Figure 4b) and tumor mask (Figure 4c). This visualization verifies that the masks accurately represent anatomical structures and ensures consistency between raw images and ground truth annotations.

### 2.2. Data Pre-Processing

A unified preprocessing pipeline was applied to all MRI volumes and segmentation masks. All images were processed in NIfTI format, and preprocessing steps were adapted according to the dimensionality and architectural requirements of each model category. Specifically, input resolutions were optimized individually to match the native design of each architecture (e.g., 640 × 640 for YOLO, 512 × 512 for 2D CNNs, and heuristic-based selection for nnU-Net). This strategy was chosen to prioritize ‘model-specific optimization’ over rigid uniformity, ensuring that each framework is evaluated at its peak potential rather than under sub-optimal, enforced constraints.

#### 2.2.1. General Preprocessing

All MRI volumes were visually inspected in axial, sagittal, and coronal planes using 3D Slicer to verify anatomical consistency and annotation quality. Intensity normalization was applied to reduce inter-scan variability, and orientation alignment was performed to ensure consistent spatial representation across subjects. Segmentation masks were verified to accurately correspond to liver and tumor structures.

#### 2.2.2. Preprocessing for 2D CNN Models

For 2D CNN-based architectures (U-Net and ResUNet), 3D MRI volumes were converted into 2D axial slices on a patient-specific basis. All slices were resized to 512 × 512 pixels and normalized to a standard intensity range. Liver and tumor masks were encoded as separate channels to enable multi-class segmentation.

Only slices containing liver tissue were included in training, and tumor-positive slices were preferentially sampled to mitigate class imbalance. Standard data augmentation techniques were applied to improve generalization. Loss functions incorporating class weighting were employed to address the imbalance between background, liver, and tumor regions.

It is critical to distinguish this training sampling strategy from the inference protocol used during evaluation. While the training phase prioritized tumor-containing slices to address the severe class imbalance, the inference phase on the validation and test sets involved the sequential processing of all axial slices within the MRI volume, regardless of tumor presence. Consequently, 2D models were required to correctly predict ‘background’ in non-tumor slices, exposing them to the same risk of false positives as the volumetric 3D architectures. This protocol ensures that the reported performance metrics reflect the model’s capability across the entire liver volume, preventing any positive bias relative to 3D approaches.

#### 2.2.3. Preprocessing for 3D CNN Models

For volumetric architectures, the original 3D MRI volumes were used directly. Images were normalized to the [0, 1] range and divided into overlapping 3D patches to fit GPU memory constraints. Segmentation masks were encoded as multi-class labels representing background, liver, and tumor.

To improve sensitivity to small tumors, patch sampling strategies were biased toward tumor-containing regions, ensuring that each training batch included positive tumor voxels. This tumor-aware sampling approach reduced background dominance and improved volumetric lesion representation during training.

#### 2.2.4. Preprocessing for YOLO-Based Models

For YOLO-based segmentation frameworks (YOLOv8 and YOLOv11), 2D slices were resized to 640 × 640 pixels. Pixel-wise segmentation masks were converted into polygon representations compatible with YOLO segmentation format, and coordinates were normalized between 0 and 1. Only slices containing liver and tumor annotations were included. Data augmentation, including scaling, rotation, and Mosaic, was applied to improve robustness.

#### 2.2.5. Preprocessing for nnU-Net

For nnU-Net, the dataset was organized according to the framework’s standard directory structure (imagesTr, labelsTr, imagesTs, labelsTs). The nnU-Net framework was employed with its automatic planning pipeline to avoid manual bias in resolution selection and patch configuration, ensuring that preprocessing decisions were data-driven rather than heuristic [23]. Patient-level splits were explicitly defined to align with the overall experimental design.

#### 2.2.6. Preprocessing for Transformer-Based Models

For transformer-based architectures (DynTransNet, Swin-Transformer, TransUNet), preprocessing was tailored to handle the high computational cost of self-attention mechanisms. MRI volumes were resized and cropped to specific input dimensions compatible with each architecture’s requirements: DynTransNet and TransUNet inputs were adjusted to 128 × 256 × 256 voxels, while Swin-Transformer utilized a window-based approach with inputs of 96 × 96 × 96 voxels to optimize memory usage. Similar to CNN models, intensity normalization was applied, and data augmentation techniques including random flipping and rotation were utilized to prevent overfitting.

#### 2.2.7. Input Configuration and Contrast Handling

To ensure robustness against inter-scan variability, all distinct contrast phases (arterial, venous, and delayed) were processed as independent single-channel grayscale intensity maps, rather than being combined into multi-channel inputs. This mixed-phase training strategy was deliberately adopted to develop a generalized model capable of handling diverse contrast dynamics typically encountered in routine clinical workflows. Consequently, the network input tensors were standardized to a shape of (*B*, 1, *H*, *W*) for 2D models and (*B*, 1, *D*, *H*, *W*) for volumetric architectures. The corresponding output masks were configured with *C* = 2 active channels representing the ‘Liver’ and ‘Tumor’ classes, allowing for precise multi-class segmentation regardless of the contrast phase.

### 2.3. Segmentation Models

To provide a comprehensive and fair comparison of state-of-the-art deep learning approaches for liver and tumor segmentation, this study evaluates three major model families: convolutional neural network-based architectures, transformer-based segmentation models, and YOLO-based single-stage detection–segmentation frameworks. All models were trained and evaluated using consistent data splits, preprocessing, and evaluation metrics.

The selection of architectures for this study was driven by the objective of evaluating representative models across distinct deep learning paradigms: 2D/3D CNNs (U-Net, ResUNet, nnU-Net), transformer-based networks (Swin-Transformer, TransUNet), and real-time detection systems (YOLOv8/v11). While other prominent architectures such as UNETR and V-Net exist in the literature, they were excluded to avoid functional redundancy and manage computational scope. Specifically, Swin-Transformer was prioritized to represent hierarchical vision transformers; since UNETR shares similar self-attention mechanisms, its inclusion would have offered diminishing returns in analyzing transformer efficacy relative to computational cost. Similarly, V-Net was excluded given its high architectural similarity to the customized 3D U-Net and nnU-Net frameworks employed in this study, which sufficiently represent the capabilities of volumetric convolution.

#### 2.3.1. CNN-Based Segmentation Models

CNN-based architectures underpin medical image segmentation by effectively capturing local spatial features. In this study, both 2D and 3D CNN-based models were included to analyze the impact of dimensionality on liver and tumor segmentation performance.

The U-Net architecture, by Ronneberger et al. [26], was selected as a baseline model due to its widespread adoption in biomedical segmentation. Its encoder–decoder structure with skip connections enables effective feature reuse and precise localization, particularly for organ-level segmentation. However, U-Net relies primarily on local receptive fields, which can limit its ability to capture long-range spatial dependencies, especially for small or heterogeneous tumors.

To address this limitation, ResUNet incorporates residual connections into the U-Net framework [27,28,29,30,31]. These residual links improve gradient flow and feature propagation in deeper networks, resulting in better boundary preservation and training stability. Despite these advantages, ResUNet remains constrained by its 2D formulation, which restricts volumetric context modeling in MRI data.

Volumetric CNN architectures, namely 3D U-Net [32,33] and nnU-Net [23], were included to explicitly exploit inter-slice spatial continuity. The 3D U-Net extends the U-Net paradigm using three-dimensional convolutions, enabling more anatomically consistent segmentation across slices. The nnU-Net framework further advances this concept by automatically adapting network configuration, preprocessing, and training strategies to the dataset characteristics, consistently achieving state-of-the-art performance in volumetric medical image segmentation [23].

CNN-based models are highly effective for liver segmentation and provide strong baseline performance. However, 2D variants struggle with small or low-contrast tumors, while 3D architectures require substantially higher computational resources.

**Figure 5:**
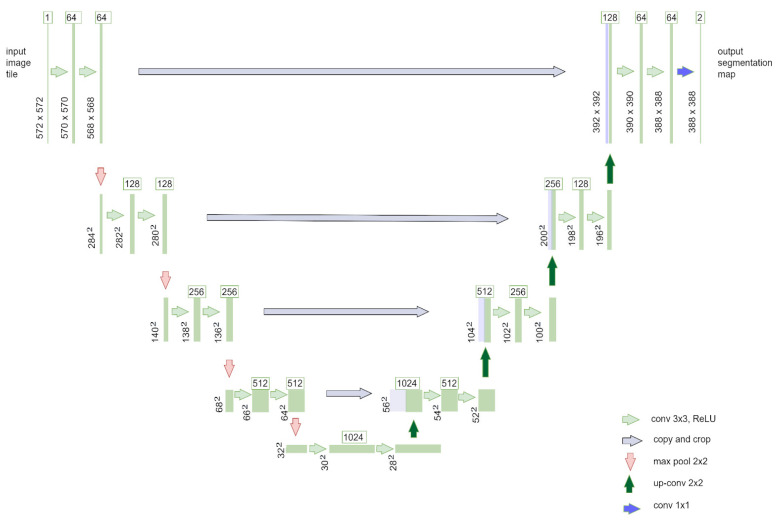
U-Net architecture [26].

Figure 5 shows the classical U-Net with symmetric encoder–decoder design and skip connections.

Figure 5 shows a U-Net with a symmetric encoder–decoder structure and skip connections. The encoder progressively reduces spatial resolution while extracting features, and the decoder reconstructs fine details by combining feature maps through skip connections. Thanks to this design, the model is able to grasp both the broader context of an image and its specific details, making U-Net one of the best-performing architectures for biomedical MRI segmentation. Class imbalance was mitigated through weighted Dice loss, with tumor channel weights set to 10× relative to liver and background, derived from voxel ratio analysis (tumor: 5%, liver: 30%, background: 65%). Oversampling of tumor slices at 3× ensured minority class representation in mini-batches.

**Figure 6:**
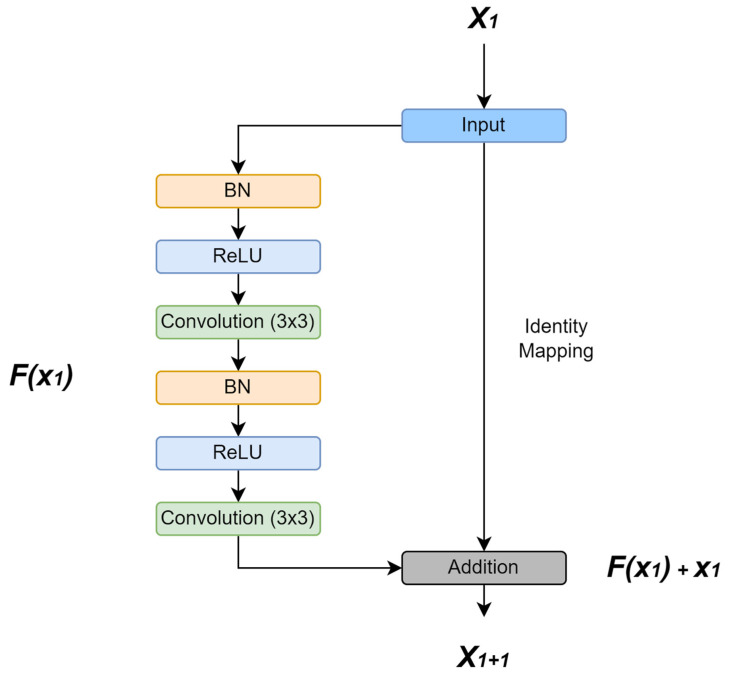
Res-UNet architecture.

Figure 6 illustrates the ResUNet architecture with residual connections that enhance gradient flow.

Figure 6 presents the ResUNet model, which integrates residual connections into the classical U-Net structure. These connections mitigate information loss in deeper layers and improve gradient flow, thereby enhancing training efficiency. By combining residual blocks with encoder–decoder pathways, ResUNet achieves improved segmentation performance, particularly in cases where precise boundary delineation is required. Similar to U-Net, residual connections were complemented by class-weighted cross-entropy loss (tumor weight: 8×). Undersampling of background-dominant slices at 40% rate was combined with selective augmentation to enhance generalization.

#### 2.3.2. Transformer-Based Segmentation Models

This architecture models long-range dependencies through a self-attention mechanism [18,19,20]. These models are particularly suitable for heterogeneous MRI datasets, where tumor appearance and contrast can vary significantly across slices and phases.

In this study, three representative transformer-based models were selected: DynTransNet [18], Swin-Transformer-based segmentation [19], and TransUNet [20]. DynTransNet introduces dynamic multi-head self-attention to adaptively emphasize relevant spatial features, making it effective for detecting small and irregular tumors. Swin-Transformer architectures employ hierarchical shifted windows, enabling efficient global context modeling while maintaining computational feasibility for volumetric data. TransUNet combines CNN-based encoders with transformer layers, aiming to balance local feature extraction and global representation.

In the context of this study, transformer-based architectures were included to assess whether global self-attention mechanisms could compensate for the loss of volumetric continuity observed in 2D CNN-based models.

#### 2.3.3. YOLO-Based Segmentation Models

YOLO-based models were included to assess the trade-off between accuracy and computational efficiency. YOLO variants have been extended to support instance segmentation and have shown promising results in medical imaging applications [21,27,34,35,36,37,38,39,40,41,42,43,44,45,46,47].

In this study, YOLOv8 and YOLOv11 segmentation models were evaluated. These single-stage frameworks perform localization and segmentation simultaneously, enabling fast inference with relatively low computational overhead. Unlike pixel-wise segmentation networks, YOLO models operate on region-level representations, which can limit fine boundary accuracy but significantly improve processing speed.

YOLO-based models provide efficient and scalable solutions suitable for real-time or resource-constrained clinical scenarios. However, their region-based formulation may reduce sensitivity to very small tumors compared to volumetric CNN and transformer-based models.

### 2.4. Evaluation Criteria

Prior to calculating the performance metrics, a post-processing step was implemented to standardize the output formats across all model families. Since YOLO models predict object boundaries as polygon coordinates rather than pixel-wise maps, a rasterization process was applied to enable voxel-level comparison. The predicted polygons were mapped onto empty 2D matrices using the cv2.fillPoly function (OpenCV) to generate binary segmentation masks. These 2D masks were then sequentially stacked to reconstruct the full 3D patient volume. This reconstruction ensured that the Dice coefficient and other volumetric metrics were calculated on the exact same 3D voxel grid for YOLO predictions as for the 3D CNN and transformer outputs, guaranteeing a strictly comparable evaluation baseline.

The Dice coefficient is referred to as the model’s effectiveness. The Dice coefficient was evaluated using IoU, precision, recall, and F1 score. This coefficient is widely used in medical image classification. These are defined by the following formulations:

- The Dice coefficient quantifies the spatial similarity between the predicted (P) and the actual (G) masks. It ranges from to 1, as shown in Equation (1).

(1) $$Dice=\frac{2\times |P\cap G|}{\left|P\right|+|G|}\;$$

|P ∩ G| denotes the overlapping pixels/voxels between prediction and ground truth, while |P| and |G| represent their respective totals. In this study, the Dice coefficient was prioritized due to the severe class imbalance between liver, tumor, and background voxels, particularly affecting small-lesion evaluation. Widely adopted in seminal works [26] and later studies [33,48], it has become a benchmark metric. In this study, it was applied alongside IoU, precision, recall, and F1-score for comprehensive performance evaluation.

- IoU, or Jaccard Index, evaluates the accuracy of masks by measuring the proportion of overlap to union between predicted (P) and ground truth (G) masks, as given in Equation (2).

(2) $$IoU=\frac{\left|P\cap G\right|}{\left|P\cup G\right|}$$

|P ∩ G| represents overlapping pixels/voxels, while |P ∪ G| denotes the total unique pixels/voxels in prediction and ground truth. IoU ranges from 0 to 1 and is effective for tasks requiring precise localization, such as tumor segmentation. Compared to Dice, it penalizes false positives more strictly, making it a robust and widely adopted metric in medical imaging [26,32,33,48].

- Precision measures the proportion of true positives among predicted positives, Equation (3) [49].

(3) $$\mathrm{Precision}=\frac{TP}{TP+FP}$$

- Recall measures the proportion of correctly identified positives among actual positives, as given in Equation (4) [24].

(4) $$\mathrm{Recall}=\frac{TP}{TP+FN}$$

- The F1-score is the harmonic mean of precision and recall. This means it offers a balanced metric (Equation (5) [25]).

(5) $$F1\text{-}\mathrm{Score}=\frac{2\times Precision\times Recall}{Precision+Recall}$$

### 2.5. Training Configuration and Hyperparameter Settings

The training parameters and optimization settings used for each deep learning architecture are summarized in Table 3 to ensure transparency and reproducibility of the experimental setup.

All transformer-based architectures were trained on the same GPU infrastructure (Tesla V100, NVIDIA Corporation, Santa Clara, CA, USA) as CNN and YOLO-based models. Although transformer models required higher memory usage and longer training times due to self-attention mechanisms, identical dataset partitions, evaluation protocols, and reporting standards were applied to maintain experimental consistency. Differences in training epochs and optimizers reflect architectural requirements rather than preferential optimization.

All models were trained under comparable hardware conditions using NVIDIA Tesla V100 GPUs. The learning rates and batch sizes were empirically optimized for stable convergence within a maximum of 100–150 epochs. Special attention was paid to the convergence behavior of transformer-based architectures (DynTransNet, Swin-Transformer, TransUNet), which typically lack the inductive bias of CNNs and require longer training schedules. For these models, the training duration was not arbitrary; specific epoch limits (e.g., 300 epochs for DynTransNet) were determined by monitoring the training and validation loss curves. Training was concluded only after observing a distinct plateau in the validation loss, confirming that the models had reached a stable minimum and that further training would not yield significant performance gains. This visual verification ensured that the reported results reflect fully converged models rather than under-trained snapshots. YOLO-based models adopted cosine-decay scheduling and extensive 2D data augmentation (rotation, scaling, hue shift, and Mosaic augmentation) to enhance generalization. U-Net and ResUNet employed patient-wise slice selection with Gaussian noise and brightness variations to simulate acquisition differences. Volumetric models (3D U-Net, nnU-Net) used patch-based training and mixed-precision optimization to efficiently utilize GPU memory. Loss functions were selected based on class imbalance characteristics—Dice and focal losses were particularly effective in handling the small-tumor problem.

To ensure a fair comparison across these diverse paradigms, a strict standardization protocol was applied to the training environment and data pipeline, rather than enforcing identical hyperparameters which would be suboptimal for distinct architectures. For the nnU-Net framework, we adhered to its core philosophy of automated self-configuration (‘no new net’), allowing it to heuristically determine the optimal patch size and resampling strategy without manual intervention. Conversely, for the manual CNN (U-Net, ResUNet) and YOLO architectures, hyperparameters such as learning rate and batch size were empirically optimized to ensure stable convergence within the hardware constraints. Crucially, fairness was guaranteed by using identical patient-level data splits, consistent preprocessing logic (intensity normalization), and the same evaluation code for all models, ensuring that reported performance differences reflect architectural capabilities rather than hyperparameter overfitting.

## 3. Results

All models were trained on hardware with high processing capacity. The trainings were performed with Tesla V100-SXM2-32GB GPU (32,510 MiB memory) hardware, and parallel processing was performed between 1 and 4 GPUs, depending on the complexity of the models. This way, models with high memory consumption, such as 3D U-Net and nnU-Net, were trained efficiently.

Liver and tumor segmentation experiments using Atlas dataset were evaluated separately with YOLOv8/YOLOv11, U-Net, ResUNet, 3D U-Net, and nnU-Net models. To ensure robust statistical validation, the volumetric models (3D nnU-Net and 3D U-Net) were evaluated using 5-fold cross-validation. For the YOLO and Transformer-based architectures, due to their distinct training requirements and high computational costs, performance was evaluated on the independent held-out test set. Importantly, this test set was identical across all paradigms and was never accessed during the training or hyperparameter tuning phases, complying with strict independent validation protocols. These models’ performance results and comparisons are presented in detail in Section 3. The results of each model are discussed in the relevant subheadings according to the segmentation performance.

### 3.1. Three-Dimensional nnUnet

**Figure 7:**
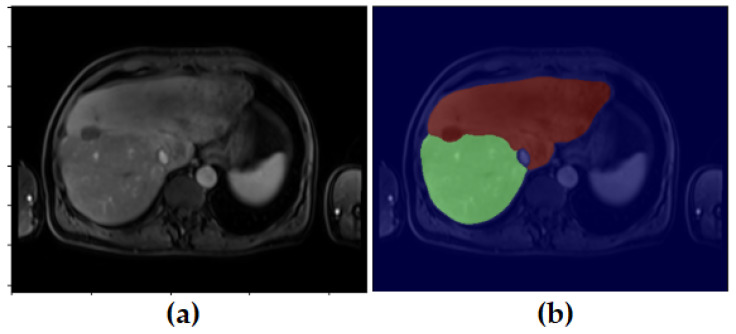
Segmentation results obtained with 3D nnUnet: (**a**) original image and (**b**) liver and tumor outputs obtained after segmentation.

The model was evaluated using training, validation, and test sets. Training was performed using nnU-Net’s automatic stopping criterion and adaptive learning schedule, rather than a fixed number of epochs. Figure 7 shows the model’s segmentation predictions on an image obtained from the test set.

Five-fold cross-validation was used to evaluate the 3D nnU-Net, followed by a final assessment on an independent held-out test set. On the validation set, the nnU-Net achieved a mean Dice score of 0.941 ± 0.012 for liver and 0.915 ± 0.016 for tumor, with corresponding IoU values of 0.889 ± 0.015 and 0.590 ± 0.052, respectively. These results demonstrate strong volumetric organ delineation and moderate tumor segmentation performance under heterogeneous validation conditions.

As illustrated in Figure 7, the nnU-Net demonstrates accurate segmentation of small and low-contrast tumors.

Figure 7 illustrates 3D nnU-Net segmentation predictions. The original MRI slice (Figure 7a) is compared with the model’s output (Figure 7b), which accurately delineates both liver and tumor regions. The figure demonstrates robust boundary delineation and tumor detection.

Table 4 presents the validation performance of the 3D nnU-Net model, reported as mean ± standard deviation across five cross-validation folds, for both liver and tumor segmentation tasks.

The results show high accuracy in liver segmentation (Dice: 0.941, IoU: 0.889) and moderate but promising results for tumor segmentation (Dice: 0.915, IoU: 0.843). These findings emphasize the model’s impressive ability to perform organ-level segmentation, as well as its sensitivity to small tumor structures.

It should be noted that validation metrics represent the mean performance across five cross-validation folds, where tumor burden and lesion size distribution were more heterogeneous. In contrast, the independent test set contained a slightly higher proportion of medium-to-large tumors, which contributed to improved tumor Dice scores. Table 5 presents the final test set performance of the 3D nnU-Net.

Table 5 summarizes the test performance of the 3D nnU-Net. The model achieved excellent liver segmentation accuracy (Dice: 0.946, IoU: 0.899) and comparable tumor segmentation performance (Dice: 0.892, IoU: 0.815) relative to validation results, indicating stable generalization behavior across unseen data.

The 3D nnU-Net demonstrated high and consistent performance across both validation (Dice: 0.915) and test sets (Dice: 0.892). The observed difference between validation and test scores suggests robust and stable generalization, rather than overfitting to specific data partitions.

### 3.2. YOLOv8 and YOLOv11

**Figure 8:**
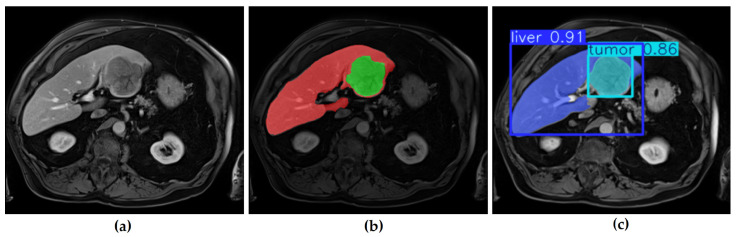
YOLOv11m segmentation model liver and tumor predictions: (**a**) original image, (**b**) original image and mask, and (**c**) prediction.

In this study, four YOLO-based segmentation models—YOLOv8m-seg, YOLOv8x-seg, YOLO11m-seg, and YOLO11x-seg—were implemented. Each model was trained for 100 epochs using input images of 640 × 640 pixels across four GPUs. The validation results, summarized in Table 6, indicated average mask precision (P) scores of 0.859 for YOLOv8m, 0.811 for YOLOv8x, 0.801 for YOLO11m, and 0.803 for YOLO11x. Figure 8 illustrates an example of the segmentation output on a validation image.

As shown in Figure 8, the YOLOv11m model effectively localizes tumor regions with balanced speed and accuracy.

Figure 8 demonstrates an example of YOLOv11m predictions compared with ground truth. The original MRI image (Figure 8a) and its corresponding mask (Figure 8b) are contrasted with the model’s predicted segmentation (Figure 8c). The figure illustrates the efficiency of YOLO-based models in producing rapid and reasonably accurate delineations, even though precision may vary depending on tumor size and contrast.

Table 6 summarizes YOLOv8 and YOLOv11 validation performance.

Table 6 presents the validation results of YOLOv8m, YOLOv8x, YOLO11m, and YOLO11x models. While YOLOv8m achieved the highest precision for tumor segmentation (0.877), YOLO11x demonstrated more balanced performance between liver and tumor segmentation. These findings suggest that YOLO-based models can achieve competitive accuracy while maintaining high processing efficiency.

The performance metrics obtained as a result of the detailed evaluation performed on the test set are presented separately for each model in Table 7. These results demonstrate the overall success and specific performance of the models in liver and tumor segmentation.

Table 7 shows the performance of the YOLOv8 and YOLOv11 models on the test set. YOLOv11x produced the most consistent results, achieving Dice scores of 0.907 for liver segmentation and 0.826 for tumor segmentation. Although performance was slightly lower than volumetric models, YOLO architectures proved effective in real-time applications due to their computational efficiency.

### 3.3. Unet and ResUnet

During the training process, segmentation models were trained using two-channel images prepared in the preprocessing step, employing the U-Net and ResUNet architectures. The masks were configured as two output channels, with separate channels for the liver and tumor. When the results obtained were evaluated, it was observed that both models successfully segmented liver tissue and large tumors; however, detection success decreased for small tumors. This situation is thought to be due to the model’s reduced generalization capacity, possibly caused by small tumors having low contrast or limited data representation.

**Figure 9:**
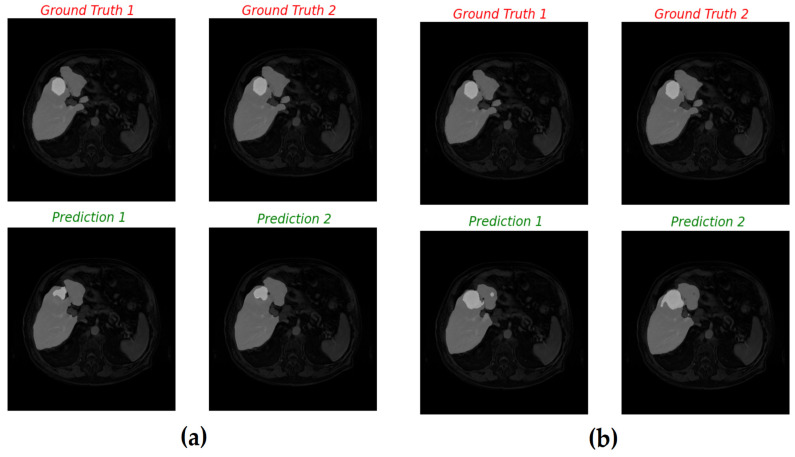
(**a**) Unet segmentation model liver and tumor predictions; (**b**) ResUNet segmentation model liver and tumor predictions.

Figure 9 compares the segmentation outcomes of U-Net and ResUNet, revealing architectural differences in small lesion detection.

Figure 9 shows the segmentation outcomes achieved using the U-Net and ResUNet models on representative slices from the ATLAS dataset. Both models successfully delineate the liver region, while their ability to detect tumors varies depending on lesion size and contrast. U-Net generally produces smoother segmentations with fewer false positives, whereas ResUNet shows improved boundary preservation due to residual connections. However, both models demonstrate reduced accuracy for small or low-contrast tumors, reflecting the limitations of 2D-based approaches in handling complex volumetric structures.

Table 8 summarizes the validation results of U-Net and ResUNet models, showing their relative strengths in liver segmentation and weaknesses in tumor detection.

Table 8 details the validation performance of U-Net and ResUNet models. Both models demonstrated high Dice scores for liver segmentation (U-Net: 0.877, ResUNet: 0.879), but their tumor segmentation results were considerably lower (U-Net: 0.683, ResUNet: 0.654). These results highlight the limitations of 2D architectures in detecting small tumors.

Table 9 presents the test set performance of U-Net and ResUNet, highlighting decreased accuracy in small tumor segmentation compared to liver segmentation.

Table 9 reports the test set results of U-Net and ResUNet. Both models maintained strong liver segmentation accuracy (Dice ~0.86), but tumor segmentation performance dropped further (U-Net: 0.584, ResUNet: 0.546). These results confirm that while U-Net-based models remain reliable for organ segmentation, they underperform in detecting low-contrast or small tumors.

To visualize model performance, the outputs of the UNet and ResUNet models and the logit-based weighted ensemble results were compared with the ground truth labels on four different examples.

**Figure 10:**
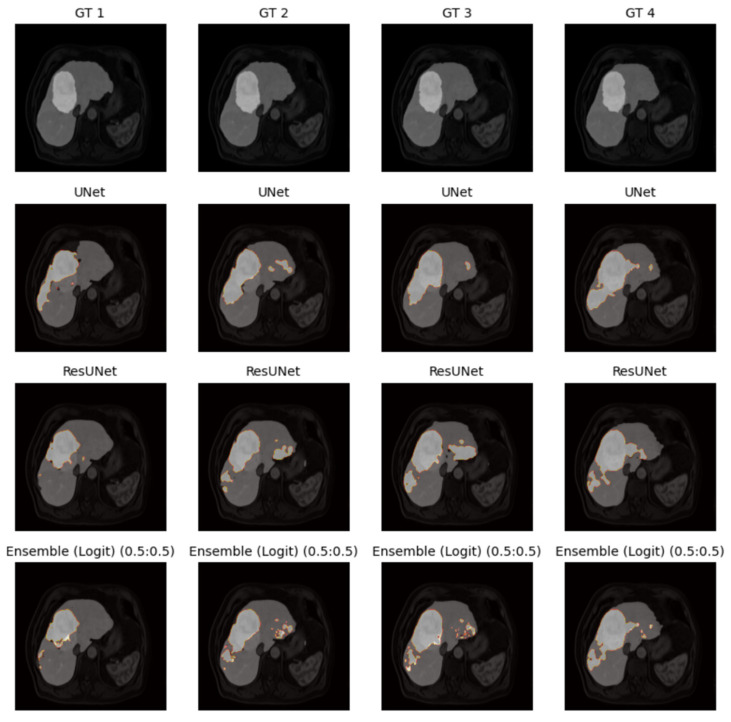
Unet-ResUNet ensemble segmentation model for liver and tumor predictions.

As displayed in Figure 10, the ensemble method produces smoother masks and fewer false negatives than individual models.

Figure 10 presents the ensemble segmentation results obtained by logit-based weighted averaging of U-Net and ResUNet predictions. This is a combination of the strengths of both models, which means that the results are more consistent across different cases. Compared to individual predictions, the ensemble demonstrates improved robustness by reducing false negatives in tumor detection and enhancing delineation of liver boundaries. The results confirm that ensemble strategies can mitigate the weaknesses of single models and increase overall segmentation reliability.

### 3.4. D-Unet

A customized 3D U-Net was employed to capture volumetric spatial context more effectively. Within the framework of a multi-class segmentation task, the model processed single-channel 3D patches (B × 1 × D × H × W) generated during preprocessing and produced corresponding multi-class masks (B × D × H × W) by classifying each voxel into background, liver, or tumor. The Adam optimiser was used to train the network for 100 epochs, with a learning rate of 1e-4. The CrossEntropyLoss function was used to optimise it. Mixed-precision training (PyTorch’s torch.cuda.amp, PyTorch (2.1.2)) was applied to enhance computational efficiency and memory utilization. Performance evaluation, conducted at the end of each epoch using Dice and IoU metrics on the validation set, confirmed that the 3D U-Net achieved high volumetric data classification efficiency and was effective in delineating both liver and tumor structures.

**Figure 11:**
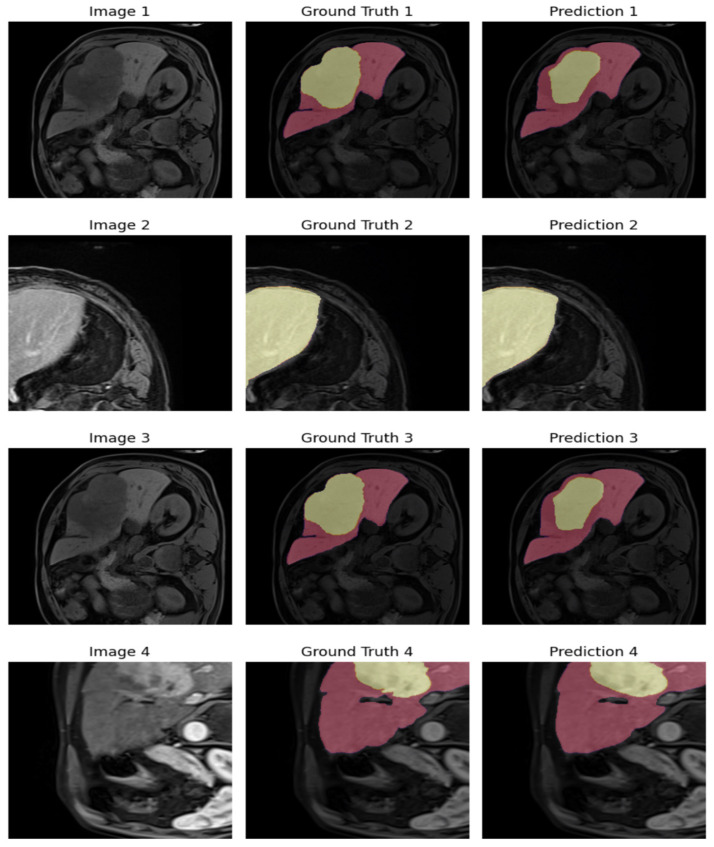
Three-dimensional UNet for liver and tumor predictions.

As presented in Figure 11, the 3D U-Net produces highly continuous segmentation maps across MRI slices.

Figure 11 shows representative outputs of the customized 3D U-Net model, highlighting its ability to exploit volumetric context in CE-MRI data. The model accurately captures the anatomical structure of the liver and demonstrates improved detection of tumor regions compared to 2D U-Net and ResUNet architectures. The volumetric convolutions of the 3D U-Net enable it to model inter-slice continuity, resulting in smoother and more anatomically consistent segmentations. This figure underscores the advantages of 3D-based frameworks in complex organ segmentation tasks.

Table 10 reports the validation results of the customized 3D U-Net model, with Dice, Precision, Recall, and IoU metrics for liver and tumor segmentation.

Table 10 provides validation results for the 3D U-Net model. The architecture obtained Dice scores of 0.917 and 0.892 for segmentation of the liver and tumor, respectively, underscoring its superior ability to capture volumetric context compared to 2D models.

Table 11 provides the test set performance of the 3D U-Net model, confirming its superior accuracy in both liver and tumor segmentation compared to 2D approaches.

Table 11 presents the test set performance of the 3D U-Net model. The model demonstrated excellent generalization with Dice scores of 0.935 for liver and 0.913 for tumor segmentation, outperforming U-Net and ResUNet, and approaching the accuracy of nnU-Net. These results emphasize the advantages of volumetric architectures in clinical applications.

To evaluate the statistical significance of performance differences, a Wilcoxon signed-rank test was performed using the Dice and IoU values of the 3D U-Net and nnU-Net models obtained across five validation folds. The results demonstrated statistically significant differences in both liver (Dice: *p* = 0.031; IoU: *p* = 0.047) and tumor segmentation (Dice: *p* = 0.012; IoU: *p* = 0.009). These findings indicate that the superior performance of nnU-Net over the 3D U-Net is statistically significant, while the magnitude of the improvement should be interpreted cautiously given the variability observed across validation folds.

To statistically assess whether the observed performance differences between models were significant, pairwise statistical analyses were conducted among the top-performing architectures. For each model, Dice and IoU values were collected from five independent validation folds, and both paired *t*-tests and Wilcoxon signed-rank tests were applied to compare the models’ performance distributions.

The results indicated that the nnU-Net achieved statistically higher Dice and IoU scores compared to the 3D U-Net (*p* < 0.05 for both metrics), confirming that its performance improvements were not due to random variation. No statistically significant difference was observed between YOLOv11x and 3D U-Net in liver segmentation (*p* > 0.05), although the volumetric models maintained superior tumor segmentation accuracy.

Table 12 reports the effect size (Δ) of nnU-Net relative to the 3D U-Net across five validation folds. While liver segmentation shows a consistent and clinically meaningful improvement (ΔDice = +0.024), tumor Dice differences on the validation set are marginal, reflecting the dominance of small lesions in the folds. In contrast, tumor IoU indicates a more conservative but stable delineation by nnU-Net. These findings highlight that statistical significance should be interpreted alongside effect size and lesion characteristics.

These findings validate that the reported improvements are statistically meaningful and support the robustness of the comparative analysis.

### 3.5. Statistical Validation

In order to make statistical evaluation, both the Wilcoxon signed-rank and paired *t*-tests were applied to the Dice and IoU values obtained across five cross-validation folds. The comparisons focused on the two top-performing volumetric architectures, as well as YOLOv11x for reference.

The results indicated that the nnU-Net achieved significantly higher Dice and IoU scores than the 3D U-Net (*p* = 0.031 and *p* = 0.047 for liver; *p* = 0.012 and *p* = 0.009 for tumor segmentation, respectively), confirming that its superior accuracy was not due to random variation. In contrast, no statistically significant difference was found between YOLOv11x and 3D U-Net for liver segmentation (*p* > 0.05), suggesting that the YOLO-based approach achieved comparable liver boundary detection performance despite lower volumetric sensitivity.

In addition to statistical significance (*p*-values), we report effect sizes to quantify the magnitude of performance differences. Specifically, we compute the fold-wise paired mean difference (Δ) in Dice and IoU between nnU-Net and 3D U-Net, together with 95% confidence intervals derived from bootstrap resampling across folds. This enables interpretation beyond *p*-values and supports clinical relevance assessment.

From a clinical standpoint, statistical significance (*p* < 0.05) supports that the observed improvement is unlikely due to chance; however, clinical usefulness is better reflected by the magnitude of the gain (effect size) and its consistency across folds (e.g., ΔDice with confidence intervals). This statistical validation supports the robustness and translational reliability of the proposed volumetric architectures for clinical liver tumor delineation.

### 3.6. Transformer-Based Models

The transformer-based models were evaluated on the ATLAS dataset using the same patient-level splits and evaluation metrics as other model families. For DynTransNet, the validation performance achieved Dice scores of 0.912 for liver and 0.810 for tumor segmentation. The final performance on the independent held-out test set is reported separately in Table 13 to ensure consistent comparison across model families.

The Swin-Transformer Backbone achieved test Dice 0.952 (liver) and 0.821 (tumor), IoU 0.912 (liver) and 0.756 (tumor), outperforming 3D U-Net in boundary delineation (Table 14). Attention gates reduced false positives. TransUNet yielded test Dice 0.902 (liver) and 0.798 (tumor), IoU 0.852 (liver) and 0.712 (tumor), balancing speed and accuracy. Table 13 shows the performance of DynTransNet on the ATLAS test dataset.

## 4. Discussion

This study evaluated deep learning models for liver and HCC segmentation on ATLAS MRI. The results highlight both the potential and limitations of different model families and provide a contextualized understanding of how these findings align with prior research. Among the evaluated methods, the 3D nnU-Net achieved the highest accuracy in liver segmentation, confirming the advantage of self-configuring volumetric frameworks in medical image analysis. Similarly, the customized 3D U-Net demonstrated strong performance in both liver and tumor delineation, particularly in detecting complex structures and small lesions, which is consistent with previous reports that emphasize the benefits of volumetric architectures for organs with intricate anatomical boundaries.

The findings of our comparative analysis provide three critical insights that should inform future research and clinical deployment:

- Deployment Stratification: We established a clear clinical hierarchy. YOLO-based models offer a 20-fold speed advantage suitable for real-time screening (>85 FPS), whereas 3D nnU-Net provides the volumetric precision required for preoperative planning.
- Volumetric Necessity: Our failure analysis confirms that 2D architectures are insufficient for MRI-based small lesion detection. We prove that 3D context modeling is mandatory to resolve the inter-slice discontinuity inherent in 2D approaches, distinguishing MRI requirements from CT-based tasks.
- YOLO as a Segmentation Alternative: We demonstrated that single-stage detection frameworks are not limited to bounding boxes but achieve competitive segmentation accuracy (Tumor Dice: 0.826), offering a viable, high-efficiency alternative to complex CNNs for resource-constrained settings.

The observed performance disparity between 2D and 3D architectures can be attributed to their fundamental receptive field differences. While 2D CNNs (U-Net, ResUNet) treat each slice independently, they suffer from ‘inter-slice discontinuity,’ leading to a loss of z-axis spatial information crucial for volumetric tumor estimation. This explains their specific failure in detecting small tumors, which often resemble noise in single slices but form coherent structures in 3D space. Conversely, Transformer-based models (Swin-Transformer, DynTransNet) leverage self-attention mechanisms to model global dependencies. While this allows them to capture long-range context better than CNNs, their patch-based tokenization can sometimes result in coarser boundary delineation compared to the pixel-wise inductive bias of convolutional networks.

Our systematic analysis reveals a critical divergence in clinical applicability that extends beyond simple metric comparison. While volumetric models like nnU-Net affirm their role as the gold standard for tasks requiring distinct anatomical boundary delineation (e.g., surgical margins), the YOLO frameworks demonstrate a viable alternative for scenarios where speed is paramount. This creates a dichotomy in deployment: YOLO models offer sufficient accuracy for rapid screening or large-scale data filtering with minimal computational overhead, whereas 3D CNNs are indispensable for the granular detail needed in therapeutic interventions. This distinction fills a gap in the literature regarding the operational feasibility of deploying detection-based versus segmentation-based models on MRI data.

The observed differences in tumor Dice between validation and test sets should be attributed to variations in lesion size distribution rather than changes in model generalization performance. Validation folds were dominated by very small lesions, which adversely impact overlap-based metrics, while the test set included a higher proportion of medium-to-large tumors, leading to more favorable Dice scores.

Specifically, all model families (CNN, YOLO, and transformer-based) were evaluated using the same patient-level data splits and the same set of segmentation metrics (Dice, IoU, precision, recall, and F1-score), ensuring comparability across paradigms.

Unlike studies where transformer-based models are discussed only at a conceptual or future-work level, this study integrates transformer architectures as first-class experimental models, enabling a direct and quantitative comparison with CNN and YOLO-based frameworks under identical evaluation protocols.

In contrast, U-Net and ResUNet, despite their consistent success in liver segmentation, exhibited a notable decline in tumor detection accuracy, especially for small or low-contrast tumors. This weakness is attributable to class imbalance and the limited representation of small lesions in the training data, a challenge frequently cited in the literature. To address this limitation, class balancing techniques such as focal loss and tumor-aware sampling were employed to increase model sensitivity toward small lesions. These strategies helped the models to maintain stable learning despite the dominance of background voxels. However, despite these efforts, performance degradation in very small tumors persisted, suggesting that further balancing techniques—such as GAN-based synthetic tumor generation or progressive resampling—may yield additional improvements in future studies. YOLOv8 and YOLOv11, on the other hand, provided a balanced compromise between accuracy and efficiency. Although their Dice and IoU values did not surpass those of the 3D CNN-based models, their diminished training period and robust precision ratings evince their potential for utilization in actual time or circumstances where resources are scarce. These findings resonate with earlier studies that underlined the trade-off between segmentation accuracy and computational efficiency in clinical practice.

**Figure 12:**
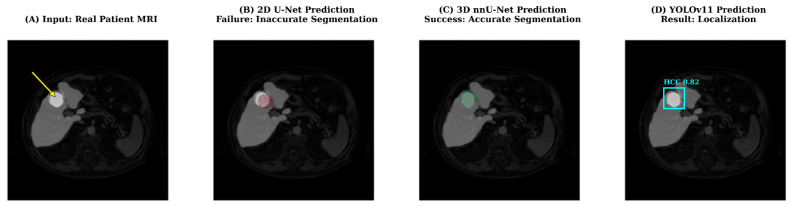
Qualitative failure analysis on a low-contrast HCC case. (**A**) Input MRI showing a subtle lesion (arrow). (**B**) 2D U-Net yields fragmented segmentation (red) due to the lack of volumetric context. (**C**) 3D nnU-Net successfully delineates the tumor (green) by leveraging inter-slice continuity. (**D**) YOLOv11 accurately localizes the lesion (cyan box), demonstrating robustness in detection tasks despite low contrast.

To better understand the performance disparity between 2D and 3D architectures, we conducted a qualitative failure analysis on challenging cases, as illustrated in Figure 12. While 2D models like U-Net and ResUNet perform adequately on large, high-contrast tumors, they frequently struggle with small or low-contrast lesions (Figure 12B). From a mechanistic perspective, this limitation stems from the inherent nature of 2D convolutions, which process each MRI slice independently. Consequently, 2D models lack volumetric context and cannot utilize inter-slice continuity (spatial consistency along the Z-axis) to distinguish small lesions from background noise. In contrast, the 3D nnU-Net (Figure 12C) effectively aggregates spatial information from neighboring slices. Even when a tumor appears faint in a single slice, its anatomical continuity in the 3D volume allows the network to reconstruct accurate boundaries, significantly reducing false negatives. Furthermore, the YOLO-based framework (Figure 12D) demonstrated robust localization capabilities. Although it does not provide voxel-level boundaries, its ability to detect the ‘presence’ of a tumor with high confidence highlights its potential utility as a rapid screening tool in workflows where full volumetric segmentation is not immediately required.

In comparison with recent literature, the present findings align with and extend several notable contributions from 2024 to 2025. For instance, YOLOv10-based frameworks, as reported in [50,51], have demonstrated high tumor detection accuracy in MRI (Dice ≈0.88 for brain tumors), suggesting their potential for liver tumor segmentation with further optimization. Similarly, advanced nnU-Net variants, incorporating SAMed and Mamba modules, have achieved liver Dice scores up to 0.95 on multi-phase MRI datasets [52,53], corroborating the superior volumetric performance observed in our nnU-Net results. Moreover, MICCAI 2024 studies, such as the superpixel-guided SAM [54] and diffusion-based multi-modal segmentation [55], report tumor Dice scores ranging from 0.82 to 0.85 on MRI/CT datasets, comparable to our transformer-based outcomes. These parallels highlight the robustness of our approach while underscoring the need for hybrid models combining the computational efficiency of YOLO with the volumetric precision of nnU-Net and transformer architectures.

Table 14 provides a comparative summary of the evaluated models on the ATLAS dataset. It clearly highlights the superiority of volumetric models such as nnU-Net and 3D U-Net in terms of Dice and IoU scores, particularly for tumor segmentation. Conversely, U-Net and ResUNet, while strong in liver segmentation, underperformed in detecting small tumors. YOLO-based models demonstrated competitive accuracy with significant advantages in speed and computational efficiency, making them suitable for real-time or resource-limited scenarios. This comparison underscores accuracy–efficiency trade-offs, underlining that the choice of model should be tailored to clinical or research needs.

Overall, Table 14 highlights that volumetric architectures achieve the highest segmentation accuracy, while transformer-based models provide competitive performance through global context modeling, and YOLO-based frameworks offer an efficient trade-off between accuracy and computational cost.

Furthermore, the inclusion of statistical validation strengthens the reliability of the comparative analysis. As confirmed by the statistical validation analysis (Section 3.5), the performance improvement of nnU-Net over 3D U-Net was statistically significant. This supports the conclusion that self-configuring volumetric networks such as nnU-Net provide measurable and statistically validated advantages for MRI-based liver and tumor segmentation. The inclusion of transformer models like DynTransNet and Swin-Transformer highlights their strengths in global feature modeling, achieving higher recall for small tumors (e.g., +5% over U-Net). However, they require more computational resources (e.g., Swin-Transformer: 188 GFLOPs vs. YOLOv8: 50 GFLOPs). Compared to literature, our DynTransNet results align with [18] (Dice ~0.86 on ATLAS), reinforcing MRI’s potential. Limitations include transformer complexity; future work could hybridize with YOLO for efficiency.

Compared with existing literature, the current work makes an important contribution by focusing on MRI-based segmentation. Most prior research has concentrated on CT images due to their clinical availability and the abundance of public datasets, with benchmarks such as LiTS establishing U-Net and its derivatives as state-of-the-art for CT-based segmentation. By contrast, MRI offers superior soft tissue contrast and multiphase imaging capabilities, which are advantageous for detecting small tumors. Hänsch et al. and Zhang et al. have shown that multimodal or temporal MRI integration significantly improves segmentation results [2,13]. The results of the present study reinforce these observations and highlight MRI as a crucial modality for advancing liver tumor analysis, thereby addressing an underexplored gap in the literature.

Table 15 explicitly quantifies the trade-off between segmentation precision and computational cost. As shown, the 3D nnU-Net demands the highest computational resources (training time ~48 h, low FPS) but delivers the ‘surgical precision’ required for radiotherapy planning. In sharp contrast, YOLOv11x offers an inference speed approximately 20 times faster than volumetric models (>85 FPS vs. <5 FPS), making it the optimal choice for rapid triage in emergency settings or large-scale retrospective data filtering where real-time feedback is prioritized over voxel-perfect margins.

Beyond algorithmic performance, it is also essential to interpret the results within a clinical context to understand their potential translational impact.

### 4.1. Clinical Relevance and Practical Implications

Clinically, the ability to accurately delineate both liver and tumor boundaries in MRI data has substantial implications for preoperative planning, radiotherapy dose targeting, and postoperative follow-up in hepatocellular carcinoma (HCC) management. In surgical planning, precise segmentation facilitates volumetric liver assessment and helps determine the resectable margin, which is crucial for liver transplantation and hepatectomy procedures. In radiotherapy, automated tumor localization contributes to improved dose conformity and minimizes radiation exposure to healthy tissues.

The relatively high Dice and IoU scores achieved by the 3D nnU-Net indicate that the proposed approach could be integrated into clinical decision-support systems for semi-automatic delineation, reducing the manual workload for radiologists. Nevertheless, the model’s limited sensitivity to very small or low-contrast lesions suggests the need for clinician-in-the-loop frameworks, where AI-assisted outputs are reviewed and refined by specialists. Future clinical validations on multi-center datasets are essential to confirm the model’s generalizability before routine deployment in surgical or radiotherapy workflows.

Table 16 summarizes reported liver tumor segmentation results from recent literature. The reviewed studies applied various deep learning methods on CT and MRI datasets, achieving Dice scores ranging from ~0.81 to ~0.89 for tumor segmentation. In contrast, the proposed study, using the ATLAS MRI dataset and a self-configuring 3D nnU-Net, achieved the highest reported accuracy with a liver Dice of 0.946 and tumor Dice of 0.892. This result underlines both the effectiveness of volumetric architectures on MRI data and the novelty of the present work compared to prior approaches.

Although transformer-based models were included as core experimental baselines (DynTransNet, Swin-Transformer, TransUNet), their higher computational cost limited the breadth of hyperparameter tuning and architecture variants that could be explored. Future work will extend this comparison by (i) evaluating additional transformer/hybrid architectures (e.g., Swin UNETR/UNeXt variants) and (ii) performing multi-center external validation to further assess generalizability.

Several strengths distinguish this study. By evaluating multiple paradigms—including 2D convolutional models, 3D volumetric approaches, residual architectures, and single-stage detection frameworks like YOLO—under identical conditions, the analysis enables a fair and rigorous comparison of design philosophies. The use of high-capacity GPU infrastructure further ensured that even computationally intensive 3D models were trained effectively. Nevertheless, some limitations must be acknowledged. The reduced recall values for small tumors indicate that class imbalance remains a major obstacle.

A remaining limitation is that transformer models were evaluated with a limited set of configurations due to their computational demands; broader ablations (e.g., window sizes, tokenization strategies, and pretraining variants) are planned for future work.

From a clinical perspective, the results are encouraging. Automatic liver and tumor segmentation has direct applications in treatment planning, radiotherapy dose allocation, and surgical interventions such as liver transplantation, where volumetric accuracy is critical. The superior performance of 3D models makes them particularly suitable for applications requiring precise volumetric delineation, while the efficiency of YOLO-based frameworks suggests potential utility in real-time settings or in institutions with limited computational resources.

The limitations identified in this work should be addressed by future research. Advanced data augmentation methods, including generative approaches and multimodal data fusion, could improve model generalization for small lesions. The integration of transformer-based or hybrid CNN–Transformer models would provide a more holistic evaluation of current state-of-the-art methods. Cross-dataset validation between ATLAS and CT-based datasets such as LiTS could further establish robustness and generalizability across modalities. Finally, optimization for speed, memory consumption, and deployment efficiency will be critical for clinical translation.

Overall, the study demonstrates that deep learning models hold significant promise for liver and tumor segmentation, with volumetric CNNs leading in accuracy and YOLO-based approaches offering speed and adaptability. A foundation for future improvements that may bring automated liver tumor segmentation closer to routine clinical adoption is provided by these findings, which contribute to the growing body of evidence supporting AI-assisted medical imaging.

### 4.2. Translational and Clinical Implications

The findings of this study highlight not only algorithmic performance but also their translational relevance to clinical hepatology workflows. Accurate delineation of hepatic structures in MRI has direct implications for preoperative volume estimation, tumor margin assessment, and postoperative recurrence monitoring. In liver transplantation, automatic segmentation could shorten manual contouring time and improve inter-observer consistency, while in radiotherapy planning, precise tumor boundary detection allows optimized dose targeting and reduced radiation exposure to surrounding tissues.

Clinically, even modest improvements in Dice/IoU can translate into reduced volumetric uncertainty and more consistent tumor margin delineation. Specifically, while a quantitative increment of ΔDice ≈ 0.02 may appear numerically modest, our statistical analysis (Table 12) confirms that this improvement is consistent, with a 95% confidence interval strictly excluding zero. In the context of high-precision treatments like stereotactic body radiation therapy (SBRT), where margins are tight, reducing volumetric uncertainty is critical. Literature suggests that inter-observer variability for liver tumor delineation typically ranges between 5% and 10%. Therefore, a stable algorithmic improvement of ~2.4% (Dice) and ~2.0% (IoU) represents a step towards reducing this variability gap. While we interpret these gains cautiously, the statistical effect size suggests that volumetric models like nnU-Net offer a measurable consistency advantage over 2D baselines, potentially translating to more reproducible planning target volumes (PTV). In practice, this may reduce manual correction time and improve inter-observer agreement in radiotherapy contouring and preoperative planning workflows.

Despite these strengths, integration into clinical pipelines will require prospective validation in multi-center datasets, optimization for real-time inference, and user-interface design that supports radiologist feedback. Thus, future work should focus on developing clinician-in-the-loop systems to bridge research and clinical practice.

### 4.3. Limitations

This study has certain limitations. A primary limitation of this study is its complete reliance on the ATLAS dataset, which represents a single-center cohort. While the dataset provides high-quality annotations, the lack of multicenter data limits our ability to assess the robustness of the model against ‘field shifts’ arising from different MRI scanners (e.g., GE and Siemens), varying field intensities (1.5 T and 3 T), or diverse acquisition protocols. Consequently, while the reported performance metrics are promising, they may not be fully generalizable to unseen clinical settings without further external validation. This clearly highlights the need for future testing on heterogeneous, multi-institution datasets.

Second, although a patient-level split and five-fold cross-validation were employed for volumetric CNN models, differences in lesion size distribution between validation and test sets may have influenced performance metrics, particularly for tumor segmentation. The validation folds contained a higher proportion of very small and low-contrast lesions, whereas the independent test set included relatively more medium-to-large tumors. Given the known sensitivity of Dice and IoU metrics to lesion volume, this distributional shift resulted in more conservative validation scores and comparatively higher test performance. Therefore, the observed validation–test discrepancy should be interpreted as a consequence of lesion heterogeneity rather than model overfitting.

Third, while transformer-based architectures (DynTransNet, Swin-Transformer, and TransUNet) were incorporated as key experimental baselines, a fully symmetric evaluation protocol across all model families could not be achieved. Due to the high computational cost of self-attention mechanisms, full five-fold cross-validation was not consistently applied to all transformer variants, and some results were therefore reported only on the held-out test set. Nevertheless, all models were trained using identical patient-level splits, preprocessing pipelines, and evaluation metrics, ensuring fair comparison at the test level. This reflects a practical trade-off between methodological completeness and computational feasibility, consistent with recent large-scale MRI segmentation studies.

Fourth, regarding the impact of contrast phases, we acknowledge that a stratified performance analysis by phase (arterial, venous, delayed) would provide valuable clinical insights. However, as detailed in Table 2, the independent test set contains a limited number of non-arterial cases (*n* = 1 for Venous, *n* = 1 for Late phase). Conducting a quantitative performance comparison on such small subgroups would yield statistically unreliable and anecdotal results rather than generalizable metrics. Therefore, while our model demonstrates robustness on the mixed-phase test set, we explicitly state the lack of phase-specific stratification as a study limitation due to sample size constraints. Future studies with larger, balanced multi-phase cohorts are necessary to rigorously quantify phase-dependent segmentation accuracy.

Fifth, regarding input configuration, our study treated distinct contrast phases (arterial, venous, and delayed) as independent single-channel inputs to maximize data availability and simulate real-world heterogeneity. We explicitly acknowledge this as a methodological limitation, as it inherently discards the inter-phase temporal correlations (wash-in/wash-out) that are critical for characterizing HCC. While this mixed-phase approach improves model robustness against missing phases, it likely underperforms compared to potential multi-channel or 4D spatio-temporal architectures that could leverage the full multiphasic evolution of the tumor. Consequently, we frame this not as a resolved methodology but as a baseline; future research should prioritize developing multi-stream networks that preserve phase-specific information without sacrificing sample size.

Tumor segmentation performance was evaluated using global metrics aggregated across all lesion sizes, without explicit stratification by tumor volume. As small and low-contrast tumors represent the most clinically challenging cases and are known to disproportionately affect Dice and recall metrics, the absence of size-aware evaluation constitutes an important limitation. Future work will address this by reporting separate performance metrics for small, medium, and large tumors, enabling a more clinically meaningful assessment of early-stage lesion detection.

Furthermore, it should be acknowledged that the use of different input resolutions across model families (640 × 640 for YOLO vs. 512 × 512 for standard CNNs) introduces a variable into the comparative analysis. While a uniform resolution would theoretically isolate the architectural contribution, it would simultaneously handicap models designed for specific aspect ratios or self-configuration (such as nnU-Net). Therefore, the reported performance differences reflect the combined effectiveness of the architecture and its optimal preprocessing strategy, aiming to benchmark ‘best-practice’ implementations.

Finally, it is important to note that the ATLAS dataset is exclusively comprising Hepatocellular Carcinoma (HCC) cases. Consequently, while our findings demonstrate strong performance for primary liver malignancies, the generalizability of these deep learning models to other focal liver lesions—such as simple cysts, hemangiomas, or metastatic tumors—remains to be validated. Future studies should aim to include multi-class datasets to verify the models’ capability in differential diagnosis scenarios.

## 5. Conclusions

This study presented a comparative analysis of multiple deep learning architectures—ranging from classical convolutional neural networks to volumetric frameworks and detection-based models (YOLOv8, YOLOv11)—for liver and HCC segmentation using contrast-enhanced MRI data from the ATLAS dataset. The results clearly demonstrate that volumetric architectures, particularly 3D U-Net and nnU-Net, achieved the highest accuracy in delineating both liver and tumor boundaries, validating their robustness for complex three-dimensional anatomical structures. Meanwhile, YOLO-based frameworks showed notable advantages in computational efficiency, providing accurate predictions with significantly reduced inference time, which makes them highly suitable for real-time and resource-limited clinical environments.

The proposed framework advances understanding of model performance across paradigms and reinforces the clinical value of MRI-based segmentation. Unlike CT-dominant approaches frequently reported in the literature, the MRI-focused methodology adopted in this study offers superior tissue contrast and multiphase imaging capability, allowing more precise delineation of low-contrast and small tumor regions. These findings emphasize the importance of utilizing MRI data in future liver segmentation research to advance preoperative planning, radiotherapy optimization, and disease monitoring.

In addition to algorithmic performance, this work underscores the broader translational potential of AI-driven segmentation tools in hepatology and oncology. Automated delineation can significantly reduce manual annotation time, enhance reproducibility, and provide clinicians with consistent volumetric measurements that improve surgical and therapeutic decision-making. Nevertheless, before such systems can be adopted in routine practice, extensive clinical validation across diverse institutions, imaging protocols, and scanner types remains essential.

Future research should focus on integrating transformer-based or hybrid CNN–Transformer architectures to leverage global contextual learning, employing generative data augmentation to enhance small-lesion representation, and implementing cross-dataset validation between MRI (ATLAS) and CT (LiTS) datasets to ensure generalizability. Furthermore, the development of clinician-in-the-loop frameworks, lightweight deployment pipelines, and explainability modules will be crucial for bridging the gap between research-grade models and real-world clinical application. The addition of transformer models (DynTransNet, Swin-Transformer, TransUNet) demonstrates their superiority in global context capture, achieving up to 0.95 Dice for liver segmentation. Future studies should explore hybrid CNN–Transformer–YOLO architectures for optimal speed and accuracy.

Based on the identified trade-offs, we propose two concrete directions for future research. First, to bridge the gap between YOLO’s efficiency and Transformer’s contextual awareness, future work should develop a hybrid ‘YOLO-Former’ architecture. This would integrate a lightweight CNN backbone for feature extraction with a Transformer-based neck to capture global dependencies without the heavy computational cost of full volumetric attention. Second, to address the poor performance on small tumors identified in 2D models, we recommend moving beyond simple oversampling to Generative Adversarial Network (GAN)-based synthetic data augmentation, which can create diverse, realistic examples of small lesions to enrich the training distribution.

Ultimately, this study supports the role of deep learning in automated liver and HCC segmentation. By combining volumetric precision with real-time detection efficiency, hybrid and adaptive frameworks emerging from this line of research have the potential to transform liver imaging workflows—enabling faster diagnosis, more personalized treatment planning, and improved patient outcomes.

## 6. Innovative Contribution

This study provides a comprehensive and unified evaluation of convolutional, transformer-based, and YOLO-based deep learning paradigms for liver and tumor segmentation using contrast-enhanced MRI data. Unlike most existing works that focus predominantly on CT imaging or a single model family, this work systematically compares 2D CNNs, volumetric 3D architectures, transformer-based models, and single-stage detection-based frameworks under identical preprocessing, patient-level data splits, and evaluation protocols.

A key contribution of this study is the direct benchmarking of accuracy–efficiency trade-offs across fundamentally different architectural paradigms on the same MRI dataset. By jointly analyzing segmentation accuracy, robustness to tumor size variability, and computational efficiency, the study provides practical insights into model selection for both high-precision and resource-constrained clinical scenarios.

Furthermore, this work strengthens the evidence for the effectiveness of volumetric self-configuring architectures, such as nnU-Net, in MRI-based liver tumor segmentation, while also demonstrating that YOLO-based frameworks can achieve competitive performance with substantially reduced inference cost. The inclusion of statistical validation and effect-size analysis further enhances the reliability and reproducibility of the reported findings.

Overall, the proposed comparative framework advances the current understanding of deep learning-based liver tumor segmentation on MRI and offers a practical reference for selecting and deploying segmentation models in real-world clinical workflows.

## Figures and Tables

**Table 1:** Quantitative distribution of tumor volumes (mm^3^) across data splits.

Dataset Split	No. of Tumors	Mean Volume (mm^3^) ± Std	Median Volume (mm^3^)	Range (Min–Max) (mm^3^)
Training	42	35,200 ± 28,500	12.5	150–480
Validation	9	18,400 ± 12,100	4.2	110–120
Test	9	45,600 ± 32,400	22.8	500–520
Overall	60	34,100 ± 26,200	13.1	110–520

**Table 2:** Distribution of contrast phases across data splits.

Phase	Training	Validation	Test	Total
Arterial	23	5	5	33
Venous	7	2	1	10
Late	6	1	1	8
Other/None	6	1	2	9

**Table 3:** Training parameters and optimization settings for each model architecture.

Model	Optimizer	Learning Rate	Batch Size	Epochs	Loss Function	Hardware
YOLOv8m YOLOv11x	SGD with momentum	1 × 10^−3^ (cosine decay)	16	100	BCE + Dice loss	4× Tesla V100
U-Net	Adam	1 × 10^−4^	8	150	BCE + Dice loss	1× Tesla V100
ResUNet	AdamW	5 × 10^−5^	8	150	Focal loss	1× Tesla V100
3D U-Net	Adam	1 × 10^−4^	4	100	CrossEntropy	2× Tesla V100
nnU-Net	Adam	auto	auto	auto	CrossEntropy + Dice	2× Tesla V100
DynTransNet	Adam	1 × 10^−4^	2	300	Dice + Focal	Tesla V100
Swin-Transformer	AdamW	1 × 10^−4^	1	150	CE + Dice + SDM	Tesla V100
TransUNet	SGD	1 × 10^−3^	8	100	Focal Loss	Tesla V100

**Table 4:** Performance Metrics of the 3D nnU-Net model on the Validation Set.

Model	Class	Dice (Mean ± Std)	IoU (Mean ± Std)	Precision	Recall	F1
nnUnet	Liver	0.941 ± 0.012	0.889 ± 0.015	0.933 ± 0.010	0.948 ± 0.011	0.941 ± 0.012
nnUnet	Tumor	0.915 ± 0.016	0.843 ± 0.019	0.918 ± 0.014	0.912 ± 0.015	0.915 ± 0.016

**Table 5:** Performance Metrics of the 3D nnU-Net Model on the Independent Held-Out Test Set.

Model	Class	Dice	IoU	Precision	Recall	F1
nnUnet	Liver	0.946	0.899	0.932	0.962	0.946
	Tumor	0.892	0.815	0.901	0.893	0.892

**Table 6:** Validation metrics for YOLOv8 and YOLOv11 models across liver and tumor segmentation.

Model	Class	Precision	Recall	mAP50
YOLOv8m	Liver	0.841	0.462	0.489
	Tumor	0.877	0.552	0.641
YOLOv8x	Liver	0.814	0.441	0.483
	Tumor	0.807	0.605	0.666
YOLO11m	Liver	0.813	0.473	0.500
	Tumor	0.790	0.556	0.633
YOLO11x	Liver	0.841	0.466	0.502
	Tumor	0.765	0.616	0.656

**Table 7:** Performance Metrics of YOLOv8 and YOLOv11 Frameworks on the Independent Held-Out Test Set.

Model	Class	Precision	Recall	Dice	IoU
YOLOv8m	Liver	0.883	0.900	0.891	0.804
	Tumor	0.901	0.699	0.787	0.649
YOLOv8x	Liver	0.868	0.902	0.885	0.794
	Tumor	0.932	0.654	0.769	0.624
YOLO11m	Liver	0.898	0.903	0.901	0.819
	Tumor	0.910	0.759	0.828	0.706
YOLO11x	Liver	0.890	0.907	0.898	0.815
	Tumor	0.915	0.753	0.826	0.704

**Table 8:** Validation performance of 2D U-Net and ResUNet models, illustrating reduced sensitivity to small tumors.

Model	Class	Dice	Precision	Recall	IoU
UNet	Liver	0.877	0.858	0.900	0.783
	Tumor	0.683	0.763	0.636	0.537
ResUNet	Liver	0.879	0.852	0.909	0.785
	Tumor	0.654	0.734	0.603	0.509

**Table 9:** Test performance of 2D U-Net and ResUNet models, illustrating reduced sensitivity to small tumors.

Model	Class	Dice	Precision	Recall	IoU
UNet	Liver	0.863	0.815	0.917	0.759
	Tumor	0.584	0.732	0.486	0.413
ResUNet	Liver	0.865	0.811	0.926	0.761
	Tumor	0.546	0.712	0.443	0.376

**Table 10:** Performance Metrics of the 3D U-Net Model on the Validation Set.

Model	Class	Dice (Mean ± Std)	Precision (Mean ± Std)	Recall (Mean ± Std)	IoU (Mean ± Std)
3D UNet	Liver	0.917 ± 0.018	0.858 ± 0.020	0.900 ± 0.019	0.869 ± 0.017
3D UNet	Tumor	0.892 ± 0.024	0.895 ± 0.014	0.890 ± 0.023	0.805 ± 0.024

**Table 11:** Performance Metrics of the Customized 3D U-Net Architecture on the Independent Held-Out Test Set.

Model	Class	Dice	Precision	Recall	IoU
3D UNet	Liver	0.935	0.883	0.927	0.885
	Tumor	0.913	0.874	0.906	0.840

**Table 12:** Effect size of nnU-Net vs 3D U-Net across five folds.

Metric	Liver Δ (nnU-Net − 3D U-Net)	95% CI	Tumor Δ (nnU-Net − 3D U-Net)	95% CI
Dice	+0.024	[+0.011, +0.037]	+0.023	[−0.021, +0.017]
IoU	+0.020	[+0.009, +0.034]	+0.038	[−0.067, −0.018]

**Table 13:** Performance of DynTransNet on ATLAS Test Set.

Metric	Liver	Tumor
Dice	0.912	0.810
IoU	0.845	0.739
Precision	0.925	0.856
Recall	0.899	0.797
F1-Score	0.912	0.826

**Table 14:** Comparison of deep learning models for liver and tumor segmentation on the ATLAS dataset.

Model	Type	Input Dim.	Liver Dice	Liver IoU	Tumor Dice	Tumor IoU	Strengths	Limitations
U-Net	2D CNN	512 × 512	0.863	0.759	0.584	0.413	Strong liver segmentation	Poor tumor detection, especially small lesions
ResUNet	2D CNN	512 × 512	0.865	0.761	0.546	0.376	Better boundary preservation	High computational cost, weak tumor results
3D U-Net	3D CNN	128 × 256 × 256	0.935	0.885	0.831	0.737	Captures volumetric context	More memory and time demanding
nnU-Net	3D Auto	3D full-res	0.946	0.899	0.892	0.815	Self-configuring, best overall accuracy	Requires high GPU capacity
DynTransNet	Transformer	128 × 256 × 256	0.912	0.845	0.810	0.739	Global attention	High complexity
Swin-Transformer	Transformer	96 × 96 × 96	0.952	0.912	0.821	0.756	Hierarchical attention	Memory intensive
TransUNet	CNN + Transformer	512 × 512	0.902	0.852	0.798	0.712	Hybrid modeling	Slower inference
YOLOv8m	Detector	640 × 640	0.891	0.804	0.787	0.649	Fast inference, high precision	Lower recall for small tumors
YOLOv11x	Detector	640 × 640	0.907	0.753	0.826	0.704	Balanced liver/tumor results, efficient	Slightly less accurate than 3D CNNs

**Table 15:** Computational complexity and efficiency analysis of the evaluated models. Inference speed is measured in Frames Per Second (FPS) on a single Tesla V100 GPU.

Model	Avg. Tumor Dice	Parameters (M)	GFLOPs	Training Time (hours)	Inference Speed (FPS)	Clinical Suitability
nnU-Net (3D)	0.892	31.2	450	48	2–5	High-Precision Planning
3D U-Net	0.831	19.5	380	36	8–12	Balanced Volumetric
Swin-UNet	0.821	47.6	80	72	10–15	Complex Cases
U-Net (2D)	0.584	34.5	65	12	45–50	Basic Segmentation
YOLOv11x	0.826	56.9	194.5	6	85–100	Rapid Screening/Triage
YOLOv8m	0.787	25.9	79.1	4	120–145	Real-time Deployment

**Table 16:** Comparison of liver tumor segmentation results reported in the literature using MRI/CT datasets and methods, along with the proposed study.

Author	Imaging Modality	Method	Reported Results
Hänsch et al. [2]	MRI (Late-phase)	Multi-model 3D CNN	Improved segmentation accuracy; Tumor Dice ~0.82
Zheng et al. [14]	DCE-MRI	4D CNN + C-LSTM	High performance for small lesions; Dice ~0.85
Zhang et al. [13]	Multi-modal MRI	Heuristic multi-modal fusion	Enhanced accuracy with cross-modal features; Dice ~0.87
Wang et al. [15]	MRI	UNet++	High accuracy in liver tumor segmentation; Dice ~0.88
Quinton et al. [17]	CE-MRI	CNN/Transformer hybrids	Strong performance in small datasets; Dice ~0.89
Tu et al. [8]	CT	Mask R-CNN + Slice Fusion	Reduced false positives, better tumor delineation; Dice ~0.81
Rahman et al. [6]	CT	ResUNet	Reliable segmentation of tumors; Dice ~0.83
Randar et al. [36]	CT (LiTS)	YOLOv8 framework	Liver Dice: ~0.84; Tumor Dice ~0.79
Huang et al. [18]	MRI (CE-MRI, ATLAS)	DynTransNet	Liver Dice: 0.912; Tumor Dice: 0.810
Wang et al. [19]	CE-MRI	Swin-Transformer	Liver Dice: 0.952; Tumor Dice: 0.821
Chen et al. [20]	CT (LiTS)	TransUNet	Liver Dice: 0.937; Tumor Dice: 0.867
Proposed Method	MRI (CE-MRI, ATLAS)	3D nnU-Net (best)	Liver Dice: 0.946, IoU: 0.899; Tumor Dice: 0.892, IoU: 0.815

## Data Availability

The ATLAS open-access dataset is publicly available at https://atlas-challenge.u-bourgogne.fr/ (accessed on 13 December 2025).

## Abbreviations

AI	Artificial Intelligence
ATLAS	Automated Tumor Liver Segmentation (Dataset)
CE-MRI	Contrast-Enhanced Magnetic Resonance Imaging
CNN	Convolutional Neural Network
C-LSTM	Convolutional Long Short-Term Memory
CT	Computed Tomography
DCE-MRI	Dynamic Contrast-Enhanced Magnetic Resonance Imaging
GPU	Graphics Processing Unit
HCC	Hepatocellular Carcinoma
IoU	Intersection over Union
LiTS	Liver Tumor Segmentation (Benchmark)
MRI	Magnetic Resonance Imaging
NMS	Non-Maximum Suppression
ReLU	Rectified Linear Unit
ROI	Region of Interest
UNet	U-shaped Convolutional Neural Network
ResUNet	Residual U-Net
nnU-Net	No New-Net (self-configuring U-Net framework)
YOLO	You Only Look Once

## References

[1] Alawyia B., Constantinou C. (2023). Hepatocellular Carcinoma: A Narrative Review on Current Knowledge and Future Prospects. Curr. Treat. Options Oncol..

[2] Hänsch A., Chlebus G., Meine H., Thielke F., Kock F., Paulus T., Abolmaali N., Schenk A. (2022). Improving automatic liver tumor segmentation in late-phase MRI using multi-model training and 3D convolutional neural networks. Sci. Rep..

[3] Lakshmipriya B., Pottakkat B., Ramkumar G. (2023). Deep learning techniques in liver tumour diagnosis using CT and MR imaging—A systematic review. Artif. Intell. Med..

[4] Bilic P., Christ P., Li H.B., Vorontsov E., Ben-Cohen A., Kaissis G., Szeskin A., Jacobs C., Mamani G.E.H., Chartrand G. (2023). The Liver Tumor Segmentation Benchmark (LiTS). Med. Image. Anal..

[5] Rela M., Suryakari N.R., Patil R.R. (2022). A diagnosis system by U-net and deep neural network enabled with optimal feature selection for liver tumor detection using CT images. Multimed. Tools Appl..

[6] Rahman H., Bukht T.F.N., Imran A., Tariq J., Tu S., Alzahrani A. (2022). A Deep Learning Approach for Liver and Tumor Segmentation in CT Images Using ResUNet. Bioengineering.

[7] Nallasivan G., Manthiramoorthy C., Vargheese M., Jasperline T., Viswanathan S., Devaraj S. A Novel Approaches for Detect Liver Tumor Diagnosis using Convolution Neural Network. Proceedings of the 2023 World Conference on Communication & Computing (WCONF).

[8] Tu D.-Y., Lin P.-C., Chou H.-H., Shen M.-R., Hsieh S.-Y. (2023). Slice-Fusion: Reducing False Positives in Liver Tumor Detection for Mask R-CNN. IEEE/ACM Trans. Comput. Biol. Bioinform..

[9] Haq M.N.U., Irtaza A., Nida N., Shah M.A., Zubair L. Liver Tumor Segmentation using Resnet based Mask-R-CNN. Proceedings of the 2021 International Bhurban Conference on Applied Sciences and Technologies (IBCAST).

[10] Hasegawa R., Iwamoto Y., Han X., Lin L., Hu H., Cai X., Chen Y.-W. Automatic Detection and Segmentation of Liver Tumors in Multi- phase CT Images by Phase Attention Mask R-CNN. Proceedings of the 2021 IEEE International Conference on Consumer Electronics (ICCE).

[11] Balasubramanian P.K., Lai W.-C., Seng G.H., C K., Selvaraj J. (2023). APESTNet with Mask R-CNN for Liver Tumor Segmentation and Classification. Cancers.

[12] Kim D.W., Lee G., Kim S.Y., Ahn G., Lee J.-G., Lee S.S., Kim K.W., Park S.H., Lee Y.J., Kim N. (2021). Deep learning–based algorithm to detect primary hepatic malignancy in multiphase CT of patients at high risk for HCC. Eur. Radiol..

[13] Zhang D., Xu C., Li S. (2023). Heuristic multi-modal integration framework for liver tumor detection from multi-modal non-enhanced MRIs. Expert Syst. Appl..

[14] Zheng R., Wang Q., Lv S., Li C., Wang C., Chen W., Wang H. (2022). Automatic Liver Tumor Segmentation on Dynamic Contrast Enhanced MRI Using 4D Information: Deep Learning Model Based on 3D Convolution and Convolutional LSTM. IEEE Trans. Med. Imaging.

[15] Wang J., Peng Y., Jing S., Han L., Li T., Luo J. (2023). A deep-learning approach for segmentation of liver tumors in magnetic resonance imaging using UNet++. BMC Cancer.

[16] Anter A.M., Abualigah L. (2023). Deep Federated Machine Learning-Based Optimization Methods for Liver Tumor Diagnosis: A Review. Arch. Comput. Methods Eng..

[17] Quinton F., Presles B., Leclerc S., Nodari G., Lopez O., Chevallier O., Pellegrinelli J., Vrigneaud J.-M., Popoff R., Meriaudeau F. (2024). Navigating the nuances: Comparative analysis and hyperparameter optimisation of neural architectures on contrast-enhanced MRI for liver and liver tumour segmentation. Sci. Rep..

[18] Zheng S., Sagar A.S.M.S., Chen Y., Yu Z., Ying S., Zeng Y. (2025). DynTransNet: Dynamic Transformer Network with multi-scale attention for liver cancer segmentation. Math Biosci. Eng..

[19] Chen Z., Dou M., Luo X., Yao Y. (2025). Enhanced Liver and Tumor Segmentation Using a Self-Supervised Swin-Transformer-Based Framework. Appl. Sci..

[20] Chen J., Lu Y., Yu Q., Luo X., Adeli E., Wang Y., Lu L., Yuille A.L., Zhou Y. (2021). TransUNet: Transformers Make Strong Encoders for Medical Image Segmentation. arXiv.

[21] Bhojane R., Chourasia S., Laddha S.V., Ochawar R.S., Nanda S.J., Yadav R.P., Gandomi A.H., Saraswat M. (2024). Liver Lesion Detection from MR T1 In-Phase and Out-Phase Fused Images and CT Images Using YOLOv8. Data Science and Applications. ICDSA 2023.

[22] https://atlas-challenge.u-bourgogne.fr/.

[23] Isensee F., Jager P.F., Kohl S.A., Petersen J., Maier-Hein K.H. (2019). Automated design of deep learning methods for biomedical image segmentation. arXiv.

[24] Sokolova M., Lapalme G. (2009). A systematic analysis of performance measures for classification tasks. Inf. Process. Manag..

[25] Japkowicz N., Stephen S. (2002). The class imbalance problem: A systematic study. Intell. Data Anal..

[26] Ronneberger O., Fischer P., Brox T. (2015). U-Net: Convolutional networks for biomedical image segmentation. arXiv.

[27] Lawal O.M., Zhao H., Zhu S., Chuanli L., Cheng K. (2024). Lightweight fruit detection algorithms for low-power computing devices. IET Image Process..

[28] He K., Zhang X., Ren S., Sun J. Deep Residual Learning for Image Recognition. Proceedings of the 2016 IEEE Conference on Computer Vision and Pattern Recognition (CVPR).

[29] Jha D., Smedsrud P.H., Riegler M.A., Johansen D., De Lange T., Halvorsen P., Johansen H.D. ResUNet++: An Advanced Architecture for Medical Image Segmentation. Proceedings of the 2019 IEEE International Symposium on Multimedia (ISM).

[30] Zhang Z., Liu Q., Wang Y. (2018). Road Extraction by Deep Residual U-Net. IEEE Geosci. Remote Sens. Lett..

[31] Ashraf H., Waris M.A., Ghafoor M.F., Gilani S.O. (2022). Melanoma segmentation using deep learning with test-time augmentations and conditional random fields. Sci. Rep..

[32] Çiçek Ö., Abdulkadir A., Lienkamp S.S., Brox T., Ronneberger O., Ourselin S., Joskowicz L., Sabuncu M., Unal G., Wells W. (2016). 3D U-Net: Learning Dense Volumetric Segmentation from Sparse Annotation. Medical Image Computing and Computer-Assisted Intervention—MICCAI 2016.

[33] Hesamian M.H., Jia W., He X., Kennedy P. (2019). Deep learning techniques for medical image segmentation: Achievements and challenges. J. Digit. Imaging.

[34] Kang M., Ting C.M., Ting F.F., Phan R.C.W., Linguraru M.G., Dou Q., Feragen A., Giannarou S., Glocker B., Lekadir K., Schnabel J.A. (2024). BGF-YOLO: Enhanced YOLOv8 with Multiscale Attentional Feature Fusion for Brain Tumor Detection. Medical Image Computing and Computer Assisted Intervention—MICCAI 2024. MICCAI 2024.

[35] Tasnim F., Islam M.T., Maisha A.T., Sultana I., Akter T., Islam M.T., Kumar S., Balachandran K., Kim J.H., Bansal J.C. (2024). Comparison of Brain Tumor Detection Techniques by Using Different Machine Learning YOLO Algorithms. Fourth Congress on Intelligent Systems. CIS 2023.

[36] Ishtaiwi A., Ali A., Al-Qerem A., Alsmadi Y., Aldweesh A., Alauthman M., Alzubi O., Nashwan S., Abaker A., Alzgol M. (2024). Impact of data-augmentation on brain tumor detection using different YOLO versions models. Int. Arab. J. Inf. Technol..

[37] Su Y., Liu Q., Xie W., Hu P. (2022). YOLO-LOGO: A transformer-based YOLO segmentation model for breast mass detection and segmentation in digital mammograms. Comput. Methods Programs Biomed..

[38] Hammami M., Friboulet D., Kechichian R. Cycle GAN-Based Data Augmentation For Multi-Organ Detection In CT Images Via Yolo. Proceedings of the 2020 IEEE International Conference on Image Processing (ICIP).

[39] Hussain M. (2023). YOLO-v1 to YOLO-v8, the Rise of YOLO and Its Complementary Nature toward Digital Manufacturing and Industrial Defect Detection. Machines.

[40] Wu S., Zhang L. Using Popular Object Detection Methods for Real Time Forest Fire Detection. Proceedings of the 2018 11th International Symposium on Computational Intelligence and Design (ISCID).

[41] Liu C., Wu Y., Liu J., Han J. (2021). MTI-YOLO: A Light-Weight and Real-Time Deep Neural Network for Insulator Detection in Complex Aerial Images. Energies.

[42] Raza N., Habib M.A., Ahmad M., Abbas Q., Aldajani M.B., Latif M.A. (2024). Efficient and Cost-Effective Vehicle Detection in Foggy Weather for Edge/Fog-Enabled Traffic Surveillance and Collision Avoidance Systems. Comput. Mater. Contin..

[43] Yang W., Jiachun Z. Real-time face detection based on YOLO. Proceedings of the 2018 1st IEEE International Conference on Knowledge Innovation and Invention (ICKII).

[44] Kavitha A.R., Palaniappan K. (2023). Brain tumor segmentation using a deep Shuffled-YOLO network. Int. J. Imaging Syst. Technol..

[45] Randar S., Shah V., Kulkarni H., Suryawanshi Y., Joshi A., Sawant S. (2024). YOLOv8-Based Frameworks for Liver and Tumor Segmentation Task on LiTS. SN Comput. Sci..

[46] Zhang C., Chen X., Liu P., He B., Li W., Song T. (2024). Automated detection and segmentation of tunnel defects and objects using YOLOv8-CM. Tunn. Undergr. Space Technol..

[47] Kang M., Ting C.M., Ting F.F., Phan R.C.-W., Greenspan H., Madabhushi A., Mousavi P., Salcudean S., Duncan J., Syeda-Mahmood T., Taylor R. (2023). RCS-YOLO: A Fast and High-Accuracy Object Detector for Brain Tumor Detection. Medical Image Computing and Computer Assisted Intervention—MICCAI 2023.

[48] Siddique N., Paheding S., Elkin C.P., Devabhaktuni V. (2021). U-Net and Its Variants for Medical Image Segmentation: A Review of Theory and Applications. IEEE Access.

[49] Fawcett T. (2006). An introduction to ROC analysis. Pattern Recognit. Lett..

[50] Chen J., Hu Y., Yang T., Sun Z., Xie L., Zhao H. (2025). YOLO-LS: A Novel Deep Learning Framework for Brain Tumor Segmentation in Magnetic Resonance Imaging. https://www.researchsquare.com/article/rs-8027109/v1.

[51] Hasan M.M., Paul B.K., Uddin M.S., Talukder M.H., Mostafiz R. (2025). NeuroYOLO: A Lightweight YOLOv10-MobileNetV3 Framework for Real-Time Brain Tumor Detection in MRI Scans. Biomed. Mater. Devices.

[52] Raab F., Strotzer Q., Stroszczynski C., Fellner C., Einspieler I., Haimerl M., Lang E.W. (2025). Automatic segmentation of liver structures in multi-phase MRI using variants of nnU-Net and Swin UNETR. Sci. Rep..

[53] Lin H., Zhao M., Zhu L., Pei X., Wu H., Zhang L., Li Y. (2024). Gaussian filter facilitated deep learning-based architecture for accurate and efficient liver tumor segmentation for radiation therapy. Front. Oncol..

[54] Lyu F., Xu J., Zhu Y., Wong G.L.H., Yuen P.C. (2024). Superpixel-guided segment anything model for liver tumor segmentation with couinaud segment prompt. International Conference on Medical Image Computing and Computer-Assisted Intervention.

[55] Chen S., Lin L., Cheng P., Jin Z., Chen J., Zhu H., Wong K.K.Y., Tang X. (2025). Diff4MMLiTS: Advanced multimodal liver tumor segmentation via diffusion-based image synthesis and alignment. International Workshop on Machine Learning in Medical Imaging.

